# Management nicht-finanzieller Risiken: eine Forschungsagenda

**DOI:** 10.1007/s41471-020-00096-z

**Published:** 2020-08-07

**Authors:** Günter Franke

**Affiliations:** Universität Konstanz, 78457 Konstanz, Deutschland

**Keywords:** Nicht-finanzielle Risiken, Informationsqualität, Herausforderungen an Corporate Governance, Forschungsagenda, Non-financial risks, Information quality, Corporate governance challenges, Research agenda, D23, D81, G11, G41, M5

## Abstract

Das Management nicht-finanzieller Risiken wie ESG-, Nachhaltigkeits- und Compliance-Risiken stellt Unternehmen vor große Herausforderungen. Im Gegensatz zu finanziellen Risiken ist zu nicht-finanziellen Risiken nur wenig Information verfügbar. Dies erschwert ein effektives Management erheblich. Unternehmen haben in den letzten Jahren hohe Verluste durch nicht-finanzielle Risiken erlitten. Die Corporate Governance dieser Risiken wirft zahlreiche, ungelöste Fragen auf. Dieser Beitrag skizziert mögliche Lösungsansätze und Thesen zum Einfluss der Informationsqualität. Bei diesen Fragen fühlt sich die Praxis von den Hochschulen allein gelassen. Eine Kooperation von Praxis und Hochschule zur Lösung der Fragen eröffnet attraktive Forschungsfelder für Hochschulen. Daher zeigt dieser Beitrag auch eine Forschungsagenda für Hochschulen auf.

## Abstract (en)

The management of non-financial risks such as ESG-, sustainability- and compliance risks poses a great challenge for companies. In contrast to financial risks the information on non-financial risks is very limited. This renders management quite difficult. Companies incurred big losses due to non-financial risks in recent years. Corporate governance of these risks raises many unresolved questions. This paper delineates potential answers and hypotheses about the impact of information quality. Practitioners complain about the lack of support from academia. A cooperation of practitioners and academics to resolve these questions presents attractive research fields for academia. Thus, this paper also presents a research agenda for academia.

## Informationslücken bei nicht-finanziellen Risiken

Auslöser dieses Beitrags ist der Vorwurf der Praxis, die Wissenschaft helfe ihr kaum, nicht-finanzielle Risiken zu managen, obwohl ein großer Bedarf an wissenschaftlicher Unterstützung besteht. Die Wissenschaft ziehe es vor, wohldefinierte Fragen zu klären anstelle von solchen, bei denen ein erheblicher Informationsmangel Antworten erschwert. Unter nicht-finanziellen Risiken werden in der Praxis Risiken verstanden, für die es wenig Information gibt, insbesondere keine verlässlichen Wahrscheinlichkeitsverteilungen. Dazu gehören operationale Risiken. Beispiele sind Compliance-Risiken, also Risiken aus Fehlverhalten von Mitarbeitern^1^, Cyber-Risiken und Geldwäsche-Risiken^2^. Neuerdings treten Nachhaltigkeitsrisiken hinzu^3^. Diese Risiken werden später detaillierter beschrieben. Sie treffen alle Unternehmen, insbesondere Banken. Höchst aktuell ist das nicht-finanzielle Covid 19-Risiko mit dramatischen gesundheitlichen und ökonomischen Schäden

Nicht-finanzielle Risiken fordern die Praxis heraus. Der Wissenschaftler, der sich diesen Fragen stellt, mag einen damit verbundenen Paradigmenwechsel fürchten. Dieser erfordert die Einarbeitung in ungewohnte Sachverhalte und eine andere Vorgehensweise der Forschung, auf die am Schluss dieses Beitrags eingegangen wird. Auch besteht die Sorge, Beiträge zu solchen Fragen nicht in erstklassigen Zeitschriften veröffentlichen zu können. Diese Sorge erweist sich jedoch mehr und mehr als unbegründet. Neues Forschungsterrain bietet auch neue Chancen. Daher möchte dieser Beitrag nicht nur mögliche Ansätze zum Management nicht-finanzieller Risiken und Thesen zur Informationsqualität präsentieren, sondern auch eine Forschungsagenda und insbesondere junge Forscher ermutigen, diese Agenda aufzugreifen.

Dieser Beitrag betritt Neuland. Er gehorcht nicht dem üblichen Muster, zu Beginn Forschungsfragen zu formulieren und dann anhand von theoretischen/empirischen Analysen Antworten abzuleiten. Stattdessen möchte dieser Beitrag Probleme aufgreifen, die die Praxis intensiv beschäftigen, und dazu neben möglichen Lösungsansätzen eine Forschungsagenda entwickeln, deren Abarbeitung viel Zeit und Kraft erfordern wird. Gleichzeitig werden Thesen zum Einfluss unterschiedlicher Informationsqualität auf das Management aufgestellt, die einer gründlichen Überprüfung bedürfen.

**Figure 1:**
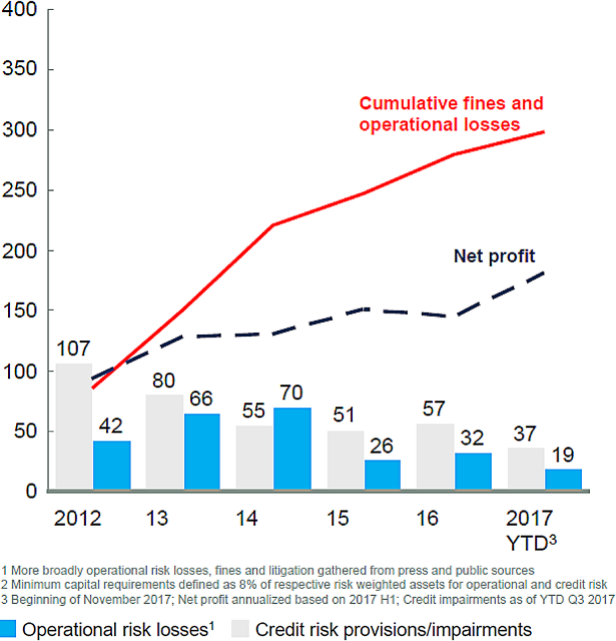

Seit der Finanzkrise 2008 wurden die Banken weltweit von operationalen Risiken, einer Teilmenge von nicht-finanziellen Risiken, hart getroffen. Schätzungen gehen von Verlusten über 400 Mrd. US-$ bis 2020 aus (Reuters 2017). Abb. 1 zeigt für die 12 größten europäischen und die 8 größten US-Banken Verluste aus operationalen Risiken wie auch Verluste und Wertberichtigungen auf Kredite. Im Zeitraum 2012 bis Anfang November 2017 belaufen sich im Jahresdurchschnitt die Verluste und Wertberichtigungen auf Kredite auf 59 Mrd. US-$, die Verluste aus operationalen Risiken auf 44 Mrd. US-$.

Diese Zahl unterschätzt allerdings die tatsächlichen Schäden. Sie misst nur Schäden aus Maßnahmen zur Beseitigung der Schadensursachen, Entschädigungen betroffener Dritter, Rechtskosten einschließlich Strafzahlungen sowie andere kürzerfristige Minderungen des Unternehmensergebnisses.

Erheblich schwerer abschätzbar sind die durch nicht-finanzielle Risiken verursachten längerfristigen Minderungen des Unternehmensergebnisses sowie Schäden anderer Stakeholder des Unternehmens; all diese werden häufig als Reputationskosten bezeichnet. Die mittel- und langfristigen Wirkungen auf Unternehmen sind oft kaum abschätzbar. Besonders deutlich wird dies in der Covid 19-Krise. Von den Banken wird dennoch erwartet, dass sie ihre Kunden großzügig mit Liquidität unterstützen. Auch wenn ein erheblicher Teil der Ausfallrisiken von der öffentlichen Hand übernommen wird, bleibt doch für jede Bank die nur schwer entscheidbare Frage, inwieweit sie den Kundenwünschen entgegenkommen kann, ohne die eigene Solvenz aufs Spiel zu setzen.

Die hohen Schäden aus nicht-finanziellen Risiken motivieren Banken, mit Nachdruck Konzepte für den Umgang mit diesen Risiken zu entwickeln. Sie hoffen dabei auf wissenschaftliche Unterstützung.

Auch wenn dieser Betrag andere Definitionen verwendet, sollen zunächst finanzielle und nicht-finanzielle Risiken^4^ gemäß dem üblichen Verständnis von Banken erläutert werden. Finanzielle Risiken sind typischerweise Marktwert- und Kreditrisiken, für die Wahrscheinlichkeitsverteilungen mithilfe ökonometrischer Analysen geschätzt werden. Nicht-finanzielle Risiken wie z. B. operationale Risiken sollten sich in den Wahrscheinlichkeitsverteilungen von Kredit- und Marktwertrisiken niederschlagen. Dies erweist sich indessen bisher als außerordentlich schwierig, weil nur wenig Information verfügbar ist.

Für finanzielle Risiken wurde in letzten 60 Jahren eine große Zahl von ökonometrischen Optimierungs- und Steuerungsmodellen entwickelt. Diese basieren auf rationalem Verhalten und anspruchsvollen Theorien wie z. B. no arbitrage-Modellen. So gibt es ausgereifte Modelle für die Bewertung von Finanzderivaten sowie ihren Einsatz für Hedging und Spekulation. Auch die Bankaufsicht nutzt solche Modelle. Dies wird in den Mindestanforderungen der BaFin für das Risikomanagement sichtbar, ebenso in den Anforderungen von EBA (European Banking Authority) und EZB.

Obwohl die finanziellen Risiken eine relativ hohe Transparenz, Berechenbarkeit und Steuerbarkeit aufweisen, benötigen die Banken dennoch anspruchsvolle Organisationen mit hoch qualifizierten Mitarbeitern, um diese Risiken zu managen. Auch sind die Banken gehalten, drei organisatorische Verteidigungslinien (3 lines of defense) zu implementieren, um Fehlsteuerungen und anderem Fehlverhalten vorzubeugen und Fehlentwicklungen rasch korrigieren zu können. Die Steuerung finanzieller Risiken haben die Banken relativ gut im Griff, auch wenn die Finanzkrise vor gut 10 Jahren gezeigt hat, dass die an die Modelle gestellten Erwartungen sich in der Krise als trügerisch herausgestellt haben.

Die Definition von finanziellen und nicht-finanziellen Risiken, wie sie bei Banken und Versicherern üblich ist, wird in diesem Beitrag modifiziert, um auch für andere Unternehmen nutzbar zu sein. Hier werden finanzielle Risiken als solche definiert, bei denen ein Unternehmen umfassende Information über die Wahrscheinlichkeitsverteilungen von Risikofaktoren und Ergebnissen besitzt. Bei nicht-finanziellen Risiken ist diese Voraussetzung nicht erfüllt^5^. Vereinfacht gesagt, finanzielle Risiken sind „rechenbar“, nicht-finanzielle nicht. Diese Einordnung geschieht indessen nicht schwarz-weiß in 1 und 0, sondern das Maß der für ein Risiko verfügbaren Information variiert von Risiko zu Risiko im Intervall [0,1]. Im Idealfall des Risikos [1] existiert für jede Handlungsalternative eine Wahrscheinlichkeitsverteilung des Ergebnisses^6^, die nicht hinterfragt wird. Alle Informationserfordernisse für eine mathematische Optimierung sind gegeben. Im schlechtesten Fall [0], den Frank Knight (1921) mit Unsicherheit benennt, verbirgt sich das Risiko hinter einer Blackbox. Da im Zeitablauf zunehmend Informationen über ein Risiko gesammelt werden, wird ein nicht-finanzielles Risiko allmählich in ein finanzielles transformiert.

Z. B. mag ein Unternehmen das Risiko bei der Einführung eines neuen Produkts im vertrauten Heimatmarkt als ein finanzielles Risiko einschätzen, während es das Risiko bei der Einführung in einem wenig vertrauten Auslandsmarkt als ein nicht- finanzielles einschätzt. Je mehr Auslandserfahrung das Unternehmen sammelt, desto mehr verschiebt sich das Einführungsrisiko von einem nicht-finanziellen zu einem finanziellen Risiko.

In der Realität bestehen häufig Mischformen von Unsicherheit und Risiko. Das Risiko eines Unternehmensportfolios wird von diversen Risikofaktoren getrieben. Häufig lässt sich für einige Risikofaktoren eine Wahrscheinlichkeitsverteilung schätzen, für andere nicht. Z. B. lassen sich Wahrscheinlichkeitsverteilungen von Wechselkursen bei „normalen“ politischen Risiken schätzen. Gleichzeitig mag es „ungewöhnliche“ politische Risiken geben, für die keine Wahrscheinlichkeitsverteilung existiert, die aber die Wahrscheinlichkeitsverteilungen der Wechselkurse beeinflussen. Gibt es für diesen typischen Mischfall von Unsicherheit und Risiko verlässliche Entscheidungs- und Organisationsmodelle?

Auch bei der Übernahme nicht-finanzieller Risiken wägt die Unternehmensleitung Ertrag und Risiko ab. Hier fehlen jedoch Wahrscheinlichkeitsverteilungen, Bewertungs- und Steuerungsmodelle weitgehend. Mitarbeiter sind gehalten, sich ein grobes Bild über die statistischen Eigenschaften nicht-finanzieller Risiken zu verschaffen. Dies können sie mit mehr oder weniger gutem Verständnis tun, so dass breiter Raum für Fehlschätzungen entsteht. Subjektive Einschätzung dominiert ihre mehr oder minder grob geschätzten Wahrscheinlichkeiten, ihre diskretionären Spielräume sind groß. Es gilt, diese konstruktiv zu nutzen. Diese Spielräume eröffnen indessen viel Raum für Verhaltensrisiken. Diese schlagen sich nieder in der Wahrnehmung dieser Risiken, in der Einschätzung der eigenen Fähigkeiten, mit diesen Risiken umzugehen, in ihren Entscheidungen und in der Überwachung der Steuerungsergebnisse. Dementsprechend wachsen die Anforderungen an die Unternehmensorganisation, um nicht-finanzielle Risiken zu managen. Nicht nur gilt es, über Anreiz- und Kontrollsysteme mögliches bewusstes oder unbewusstes Fehlverhalten von Mitarbeitern einzudämmen, sondern auch mangelhafte Information über Trial and Error-Prozesse zu verbessern, neue Information rasch in „bessere“ Entscheidungen umzusetzen und diese zu implementieren. Dies stellt hohe Anforderungen an die Fähigkeit und die Bereitschaft von Mitarbeitern, Information aufzunehmen und eigene Verhalten(smuster) anzupassen. Ebenso stellt es hohe Anforderungen an die Interaktion der Mitarbeiter und damit an die Aufbau- und Prozessorganisation.

Ein effektives Management nicht-finanzieller Risiken lässt sich nicht mit „einfachen“ Konzepten bewerkstelligen. Bei wenig verfügbarer Information tauchen zahlreiche bisher wenig erforschte Fragen der Corporate Governance auf, die in der Praxis eingehend diskutiert werden. Diese lassen sich einteilen in Planungs‑/Entscheidungsfragen und in Steuerungs- und Kontrollfragen, auch wenn eine scharfe Trennung dieser beiden Fragenkomplexe kaum möglich ist. Zur Planung/Entscheidung gehören Risikoanalyse und Risikomessung, Setzen von Prioritäten bei nicht-finanziellen Risiken, Steuerung des Risikoappetits der Entscheidungsträger, Konzepte der Entscheidungsfindung. Zur Steuerung und Kontrolle gehören die Aufbau- und Prozessorganisation. Dies schließt ein: Training von Mitarbeitern für Wahrnehmung und Beurteilung von Risiken, Schaffung einer adäquaten Risikokultur unter Einschluss von Anreizsystemen, koordinierte Umsetzung von Entscheidungen durch diverse Mitarbeiter, Kontrolle des Umsetzungserfolgs und des gemanagten Risikos, laufende Beobachtung der Umwelt, um die Information über die Risiken zu verbessern und Entscheidungen anzupassen, Verhaltenskontrolle über mehrere Verteidigungslinien.

Die Informationsdefizite sind bei verschiedenen nicht-finanziellen Risiken unterschiedlich groß, wie noch gezeigt wird. Diesen Unterschieden sollte die Corporate Governance Rechnung tragen. Dies führt zur ersten These dieses Papiers, die später erläutert wird.

### These 1:

*Je weniger Information für ein Entscheidungsproblem vorliegt, umso höher sind die Anforderungen an die Corporate Governance*.

Diese These wie auch die folgenden Thesen sind „ceteris paribus“ zu verstehen. Andere Risikofaktoren, denen das Unternehmen unterworfen ist, können den postulierten Zusammenhang überlagern, so dass eventuell andere Zusammenhänge die Beobachtungen dominieren.

In diesem Beitrag werden einige mögliche Vorgehensweisen für den Umgang mit nicht-finanziellen Risiken erörtert, ebenso werden Forschungsfragen aufgezeigt. Zudem werden Thesen über den Einfluss der verfügbaren Information auf die Corporate Governance formuliert. Umgekehrt postulieren andere Thesen Unterschiede in den Informationsanforderungen zur effektiven Lösung unterschiedlicher Teilaufgaben des Managements. Diese Thesen bedürfen einer eingehenden theoretischen und empirischen Überprüfung. Wichtige Hinweise gibt Richard Friberg mit seinem Buch: Managing risk and uncertainty – a strategic approach (2015). Er erörtert verschiedene Vorgehensweisen von Unternehmen bei wenig verfügbarer Information.

Die Verbindung von finanzwirtschaftlich konzipierten Planungs‑/Entscheidungsmodellen und Organisationskonzepten wurde bereits in den fünfziger und sechziger Jahren z. B. von Marschak (1954/55), Radner (1958/59) und Hax (1965) untersucht, später z. B. von Baldenius/Reichelstein (2006). Als sehr fruchtbar erwies sich die Agency Theorie, die ausgehend von Informationsasymmetrie optimale Anreiz- und Kontrollsysteme entwickelte. Laux (2006) resümiert zahlreiche Modelle, auch bei Delegation von Entscheidungen an mehrere Entscheidungsträger. Dabei handelt es sich stets um wohldefinierte Problemstellungen. Inzwischen existiert auch eine umfangreiche empirische Literatur zur Corporate Governance, in der meist einzelne Aspekte untersucht werden, siehe auch den Survey von Shleifer und Vishny (1997).

Der Beitrag ist wie folgt gegliedert: Im nächsten Abschnitt werden Aufgaben der Unternehmensplanung definiert und hinsichtlich der erforderlichen Information verglichen. Organisationsfragen werden am Rande angesprochen. Der dritte Abschnitt veranschaulicht Ergebnisse des zweiten Abschnitts an statischen Entscheidungsmodellen unter Einschluss von Organisationsfragen. Zudem präsentiert dieser Abschnitt Konzepte zur Vorgehensweise bei wenig verfügbarer Information und versucht, minimale Informationsanforderungen für eine verlässliche Problemlösung anzugeben. Im vierten Abschnitt werden Modelle der flexiblen Planung und Fragen des Designs der Unternehmensstrategie erörtert. Im fünften Abschnitt werden einzelne nicht-finanzielle Risiken näher beschrieben und Fragen der Corporate Governance skizziert, die in der Praxis eingehend diskutiert werden. Der letzte Abschnitt resümiert Herausforderungen für Praxis und Hochschule und präsentiert einige Gedanken zu möglichen Vorgehensweisen der Forschung.

## Aufgaben der Unternehmensplanung und Unterschiede in den Informationserfordernissen

### Operative Planung und Absicherungsmaßnahmen

Aufgabe der Unternehmensleitung ist es, ein Portfolio von Unternehmensaktivitäten zusammen zu stellen, seine Implementation laufend zu überwachen und das Portfolio entsprechend neuen Erkenntnissen anzupassen und weiter zu entwickeln. Hierbei kann die Unternehmensleitung einem engeren Fokus wie z. B. dem Shareholder Value Approach folgen oder einem breiteren Stakeholder Approach, wie er zunehmend von der Politik gefordert wird, so mit der ESG-Direktive. Ebenso gewinnen Nachhaltigkeitserfordernisse an Bedeutung, z. B. gemäß den 17 Nachhaltigkeitszielen der UN.

Die Optimierung eines Portfolios von Unternehmensaktivitäten gemäß Risiko und Ertrag gehorcht einem Portfolio-Ansatz^7^. Das Portfoliorisiko lässt sich umso effektiver managen, je zuverlässiger die stochastischen Eigenschaften von finanziellen und nicht-finanziellen Risiken wie auch die zwischen ihnen bestehenden Zusammenhänge abgeschätzt werden können. Hier liegt in Anbetracht der mageren Information über nicht-finanzielle Risiken eine große Herausforderung.

Ein evidenzbasiertes Management nicht-finanzieller Risiken startet mit einer Identifizierung und Klassifizierung dieser Risiken: In welchen Geschäftsfeldern des Unternehmens können welche Ereignisse eintreten, die die Zielerreichung des Unternehmens oder gar seine Existenz gefährden? Welcher Art können diese Ereignisse sein? Welche Risikofaktoren fördern den Eintritt solcher Ereignisse und die dadurch verursachten Schäden? Welche Schäden wurden beobachtet? Welche Evidenz gibt es von anderen Unternehmen?

Nach dem bottom up-Approach zur Fundierung einer Taxonomie nicht-finanzieller Risiken und ihrer Risikotreiber bedarf es eines top down-Approaches, der versucht, übergreifende Konzepte zum geeigneten Umgang mit nicht-finanziellen Risiken zu entwickeln und empirisch zu überprüfen. Während für den bottom up-Approach die in Unternehmen gesammelten Erfahrungen unerlässlich sind, ist der top down-Approach auch eine Aufgabe für akademische Forschung. Dabei ist eine enge Kooperation zwischen Unternehmen und Hochschulen geboten, um die notwendige Bodenhaftung akademischer Forschung zu sichern.

Das Management nicht-finanzieller Risiken setzt nicht nur die Schätzung von potentiellen Schäden aus Unternehmensaktivitäten voraus, sondern ebenso von deren Ertragspotenzial. Risiken werden in Kauf genommen, um damit Erträge zu erzielen. Zwar mag es nicht-finanzielle Risiken geben, die jenseits eines Risiko-Ertrag-Kalküls ausgeschlossen werden, wie z. B. kriminelle Aktivitäten (zero tolerance). Andere Aktivitäten des Unternehmens werden jedoch so geplant, dass damit Erträge erzielt werden, die unter Inkaufnahme des Risikos als vorteilhaft betrachtet werden. Dabei zeigt sich ein Problem, das Entscheidungen sehr erschwert. Betriebliche Vorsichtsmaßnahmen sollen helfen, potentielle Schäden aus nicht-finanziellen Risiken zu vermindern oder zu vermeiden. Vermiedene Schäden wie auch Schadensminderungen sind jedoch im Allgemeinen nicht beobachtbar. Dies erschwert nicht nur die Entscheidung über Vorsichtsmaßnahmen, sondern auch die spätere Überprüfung ihrer Wirkungen.

Die Aktivitäten eines Unternehmens lassen sich unterscheiden in operative Tätigkeiten zur Implementierung des Geschäftsmodells ebenso wie Absicherungsmaßnahmen. Diese umschließen *spezifische* Maßnahmen, mit denen Ergebnisschwankungen aus operativen Tätigkeiten (partiell) neutralisiert werden sollen, ebenso wie die *unspezifische* Absicherung von Liquidität und Solvenz durch Liquiditäts- und Kapitalreserven^8^.

Zu den spezifischen Absicherungsmaßnahmen gehören operative und finanzielle Hedgingmaßnahmen einschließlich des Abschlusses von Versicherungskontrakten.* Unspezifisches *Absicherungspotential kann durch Liquiditäts- und Kapitalreserven aufgebaut werden. Es soll helfen, potentielle finanzielle Anspannungen abzufedern und eine potentielle Insolvenz mit den zugehörigen Kosten zu vermeiden. Zudem sollen die Reserven die ungestörte Weiterführung erfolgreicher operativer Tätigkeiten ebenso wie die Umsetzung bereits geplanter längerfristiger operativer Maßnahmen auch bei ungünstigen Entwicklungen sichern.

*Unspezifische *Absicherung dient der Vorsorge gegen 1) Schäden aus nicht abgesicherten finanziellen Risiken, 2) Schäden aus nicht-finanziellen Risiken, 3) Schäden aus Risiken, die so selten eintreten, dass sie in der Planung nicht explizit erfasst werden, 4) Basisrisiken aus spezifischen Absicherungsmaßnahmen.

Dieser unternehmensinternen Perspektive steht die des Regulators/Aufsehers gegenüber. Deren Aufgabe ist es vor allem, negative externe Effekte des Unternehmensgeschehens abzuwehren. Während ein Unternehmen Liquiditäts- und Kapitalreserven gemäß seinen eigenen (internen) Kosten und Erträgen wählt, wird der Regulator/Aufseher primär externe (insbes. systemische) Kosten und Erträge, die die Allgemeinheit treffen, berücksichtigen und dabei von einer „gemeinwirtschaftlichen Wohlfahrtsfunktion“ ausgehen. Dementsprechend wird er Kapital- und Liquiditätsreserven bemessen und auf ihrer Umsetzung bestehen.

Außerdem gehören zur Absicherungspolitik weitere ex ante- und ex post-Maßnahmen, die geplant werden, *bevor* über die operativen und die Absicherungsmaßnahmen entschieden wird. Häufig wird dieses Vorgehen durch unterschiedliche Expertise der beteiligten Personen, die zudem in unterschiedlichen Abteilungen/Unternehmen arbeiten, begründet. Die vorab geplanten Maßnahmen sollen potentielle Schäden vermindern und Rahmenbedingungen für die spätere Planung von operativen und Absicherungsmaßnahmen schaffen. Unabhängig von letzteren sollten die vorab geplanten Maßnahmen vorteilhaft sein.

Ex ante-Maßnahmen sind Vorsichtsmaßnahmen, die *vor* Eintritt eines Schadensereignisses umgesetzt werden, um den Eintritt zu erschweren oder gar zu verhindern und/oder die potentielle Schadenshöhe zu vermindern.^9^ Zu den ex post-Maßnahmen zählen alle vorab geplanten Maßnahmen, die *nach *Eintritt eines Schadensereignisses umgesetzt werden, um den Schaden einzudämmen. So werden häufig vorab Notfallpläne entwickelt, die dann im Schadensfall sehr schnell umgesetzt werden. Banken z. B. müssen vorab Notfallpläne erstellen, damit bei einem Schock möglichst viele Operationen der Bank weitergeführt werden können und Bankkunden möglichst wenig geschädigt werden. Gleichzeitig sollen Notfallpläne klären, welche Mitarbeiter des Unternehmens und welche externen Personen im Schadensfall welche Aufgaben übernehmen und wie ihre effektive und rasche Zusammenarbeit gesichert wird. Da nicht für alle möglichen Schadensereignisse detaillierte Vorgehensweisen vorab geplant werden können, werden sie erst nach Beobachtung eines Schadensereignisses konkret ausgestaltet. Gleichzeitig erfordert die *spätere *Umsetzung der Notfallpläne häufig Änderungen in der Corporate Governance, die bereits *vor* Eintritt von Schadensereignissen durchgeführt werden müssen.

### Informationserfordernisse

Wie unterscheiden sich die für die verschiedenen Planungsschritte erforderlichen Informationen? Wird simultan über Tätigkeiten zur operativen Umsetzung des Geschäftsmodells und über Hedgingmaßnahmen entschieden, dann ist infolge der Portfolioeffekte oft nicht klar, welche operativen Maßnahmen der Umsetzung des Geschäftsmodells und welche der Absicherung dienen. Das ist anders bei sukzessiver Planung. Nicht selten werden im ersten Schritt operative Tätigkeiten zur Umsetzung des Geschäftsmodells beschlossen und erst danach Absicherungsmaßnahmen. Auch wenn theoretisch eine Simultanplanung einer sukzessiven vorzuziehen sein mag, so unterbleibt eine Simultanplanung in der Praxis häufig, weil 1. eine Simultanplanung komplexer als eine sukzessive ist und damit höhere administrative Kosten verursachen kann, und 2. , weil in zahlreichen Unternehmen unterschiedliche Abteilungen für die Planung operativer Maßnahmen und für die Planung von Absicherungsmaßnahmen zuständig sind. Dies gilt insbesondere für finanzielle Absicherungsmaßnahmen, die meist von der Finanzabteilung geplant werden. Diese Abteilung hat am ehesten einen Überblick über alle Risiken des Unternehmens und kann daher die Wirkungen einzelner Absicherungsmaßnahmen auf das Gesamtrisiko am besten abschätzen. Auch ist sie am besten mit den finanziellen Absicherungsinstrumenten vertraut. Um die Schwächen sukzessiver Planung abzumildern, können mehrere Planungsrunden vorgesehen werden, in denen vorläufig in einem Bereich getroffene Entscheidungen revidiert werden können, wenn sie zu erheblichen Problemen bei Entscheidungen anderer Bereiche führen.

Die Wahl eines operativen Portfolios und damit einer Unternehmensstrategie ist die anspruchsvollste Aufgabe des Managements. Je unterschiedlicher die möglichen Ergebnisse eines Portfolios sein können, umso wichtiger ist die Abschätzung der Wahrscheinlichkeitsverteilung seines Ergebnisses für eine fundierte Bewertung. Dementsprechend umfangreich sind die erforderlichen Informationen.

Sind im Rahmen einer sukzessiven Planung die Ergebnisse aus dem operativen Portfolio bereits bekannt, dann ist weniger Information für die Planung von spezifischen Absicherungsmaßnahmen erforderlich. Denn das Hedging wird nach den Differenzen zwischen den Ergebnissen aus operativem und Hedgingportfolio gesteuert. Bei hoher Hedgequalität tendieren diese Differenzen gegen null. Ihre Wahrscheinlichkeitsverteilung spielt dann nur noch eine geringe Rolle für die Absicherungsentscheidung^10^. Sind zudem die Hedgekosten niedrig, dann erweist sich ein effektives Hedging als vorteilhaft. Die Kosten *finanzieller *Hedgingmaßnahmen sind im Allgemeinen niedrig. Denn in einem vollkommenen Kapitalmarkt wäre der Marktwert dieser Maßnahmen gleich null^11^. Im Gegensatz dazu spielt die Wahrscheinlichkeitsverteilung des Ergebnisses für die Optimierung des operativen Portfolios im ersten Schritt eine größere Rolle, weil die ungehedgten operativen Ergebnisse im Allgemeinen eine erheblich größere Schwankungsbreite aufweisen. Diese Überlegungen motivieren These 2.

#### These 2:

*Die Informationserfordernisse für die Optimierung des operativen Portfolios zur Implementierung des Geschäftsmodells im ersten Schritt sind umfassender als für die Optimierung des Hedgingportfolios im zweiten Schritt. Dies gilt insbesondere für finanzielle Hedgingmaßnahmen*.

Aus ähnlichen Überlegungen folgt eine Folgethese. Werden das operative und das Hedgingportfolio gleichzeitig geplant, dann streut das Gesamtergebnis weniger als das des ungehedgten operativen Portfolios. Daher wirken sich Fehler in der Wahrscheinlichkeitsschätzung weniger gravierend aus, so dass weniger Information beschafft werden kann. Daraus folgt.

#### Folgethese 1:

*Die Informationserfordernisse für eine Simultanplanung von operativen und Hedgingmaßnahmen sind geringer als die für eine sukzessive Planung*.

Auch diese Folgethese gilt wie alle anderen Thesen ceteris paribus. Sollte mit der operativen Planung im ersten Schritt ein Lerneffekt eintreten, der die im zweiten Schritt einzusetzende Informationstechnologie ändert, dann kann dies die Informationsbeschaffung vereinfachen, aber auch komplizieren.

Eine sinnvolle Planung von Liquiditäts- und Kapitalreserve erfordert Information über die stochastischen Ergebnisse von bereits geplanten operativen und Hedgingmaßnahmen. Hierbei sind nur besonders ungünstige Ergebnisse relevant. Solche Reserven selbst werfen meist keine stochastischen Ergebnisse ab, verursachen indessen Transaktions- und Bestandshaltekosten^12^. Sind diese niedrig, dann sind auch die Kosten einer Fehlspezifizierung dieser Puffer gering. Eine zusätzliche Informationsbeschaffung zur Festlegung dieser Puffer erübrigt sich dann weitgehend. Stattdessen bietet sich eine großzügige Bemessung von Liquiditäts- und Kapitalpuffer an. Daher folgt

#### These 3:

*Bei gegebenem operativen und Hedgingportfolio ist der Vorteil zusätzlicher Information für die Festlegung von Liquiditäts- und Kapitalreserven gering, wenn die Transaktions- und Bestandshaltekosten dieser Reserven niedrig sind*.

Schäden aus Risiken, die so selten eintreten, dass sie in der Planung nicht explizit erfasst werden, beeinträchtigen These 3 nicht. Sie werden numerisch nicht abgeschätzt, so dass lediglich ein pauschaler Vorsorgebetrag gemäß der Risikokultur im Unternehmen eingeplant werden kann.

Wie beeinflussen nicht-finanzielle Risiken den Einsatz von spezifischen und unspezifischen Absicherungsmaßnahmen? Ein Versicherer kann versuchen, solche Risiken von vielen Unternehmen zu poolen und so eine effektive Diversifikation zu realisieren. Bietet er Unternehmen an, solche Risiken preisgünstig zu versichern, dann ermöglicht dies einen effektiven Hedge. Andere spezifische Absicherungsinstrumente existieren kaum, weil diese Risiken kaum „rechenbar“ sind und Dritte daher diese Risiken ohne Pooling kaum übernehmen werden. Soll bei erheblichen nicht-finanziellen Risiken das Ergebnisrisiko heruntergefahren werden, dann bietet sich oft nur eine Einschränkung der operativen Maßnahmen an. Ein Substitut für spezifische Absicherung können Liquiditäts- und Kapitalreserven sein. Sie gleichen zwar stochastische Ergebnisse der operativen Maßnahmen nicht aus, können aber die Unternehmenspolitik und -existenz wirksam stabilisieren. Damit gewinnen Liquiditäts- und Kapitalreserve an Bedeutung, wenn nicht-finanzielle Risiken zunehmen. Dies motiviert

#### These 4:

*Bei Zunahme nicht-finanzieller Risiken wachsen die Liquiditäts- und Kapitalreserven des Unternehmens, während spezifische Hedgingmaßnahmen, ausgenommen Versicherungskontrakte, an Bedeutung verlieren*.

These 4 sollte bereits bei der Planung des operativen Portfolios berücksichtigt werden. Jedes Portfolio sollte frühzeitig daraufhin überprüft werden, ob die entsprechende Reservebildung möglich und erwünscht ist. Dies setzt eine Abstimmung zwischen den Experten für nicht-finanzielle Risiken im operativen Bereich und den für die Reservebildung zuständigen Experten im Finanzbereich voraus.

## Statisches Management von Risiken

In der Entscheidungstheorie werden zahlreiche Modelle vorgeschlagen, ausgehend von umfassender Information bis hin zu verschiedenen Modellen bei eingeschränkter Information. Es geht darum, aus den Modellen bei umfassender Information „rationale“ Anhaltspunkte für Entscheidungen bei schlechterer Information zu gewinnen, so auch für das Management nicht-finanzieller Risiken. Dies erscheint wichtig, um persönlichen Idiosynkrasien oder Missverständnissen von Entscheidungsträgern bei nicht-finanziellen Risiken vorzubeugen.

In diesem Abschnitt sollen die vorangehenden Überlegungen anhand eines statischen Modells (Zwei-Zeitpunkt-Modell) veranschaulicht und dabei auch auf Organisationsfragen eingegangen werden. Als Beispiel dient ein Exporteur, der sich Wechselkurs- und Absatzrisiken gegenübersieht. Zunächst wird unterstellt, der Exporteur verfüge über umfassende Information. Sodann wird diese Prämisse aufgeweicht und gefragt, welche Modelle bei schlechterer Information infrage kommen. Schließlich wird die Frage aufgeworfen, welche minimalen Informationsanforderungen erfüllt sein müssen, damit eine Entscheidung besser fundiert ist als bei Kaffeesatzlesen.

### Entscheidung bei umfassender Information

- *Die Exportentscheidung ohne Absatzrisiko*

Ein risikoscheuer Exporteur, der lediglich ein Gut im Heimatland herstellt und in ein anderes Land exportiert, sieht sich im einfachsten Fall lediglich einem Risiko gegenüber, dem Wechselkursrisiko. Zunächst wird vom Absatz(mengen)risiko abstrahiert. Der Exporteur kennt die Wahrscheinlichkeitsverteilung des Wechselkurses. Damit ist das Wechselkursrisiko ein finanzielles Risiko. Im Ausgangsfall wählt er lediglich die Exportmenge x, um seinen erwarteten Nutzen zu maximieren. Produktions- und Exportmenge stimmen überein. Hierzu existiert eine umfangreiche Literatur (z. B. Benninga, Eldor und Zilcha 1985, Adam-Müller 1995).

Sei w der Wechselkurs im Zeitpunkt 1, definiert als Einheiten in Heimatwährung pro Einheit Fremdwährung, K_f_ die fixen Kosten in Heimatwährung, k(x) die variablen Stückkosten in Heimatwährung mit k′(x) > 0, *p*(x) der Exportpreis in Fremdwährung mit p′(x) < 0 und C der im Zeitpunkt 1 anfallende Cashflow in Heimatwährung. u(C) sei die konkave Nutzenfunktion des Cashflow C(x). Dann lautet das Entscheidungsproblem im Zeitpunkt 0, wenn Hedging ausgeschlossen ist:

$$\underset{\mathrm{x}}{\max} \mathrm{E}(\mathrm{u}(\mathrm{C}(\mathrm{x})))\quad\text{mit }\mathrm{C}(\mathrm{x})=-\mathrm{K}_{\mathrm{f}}+(\mathrm{p}(\mathrm{x})\mathrm{w}-\mathrm{k}(\mathrm{x}))\mathrm{x}$$

Für eine fundierte Entscheidung benötigt der Exporteur im Zeitpunkt 0 die Wahrscheinlichkeitsverteilung des Wechselkurses. Auch wenn er ihre Daten kauft, braucht er qualifizierte Mitarbeiter, die die Daten auf Schätzfehler prüfen sowie Risiko und Ertrag verschiedener Exportmengen anhand des Erwartungsnutzens abwägen. Schätzen die Mitarbeiter selbst die Verteilung des Wechselkurses, so brauchen sie zusätzliche Qualifikationen. Intuitionsgemäß lässt sich zeigen, dass der Exporteur bei gegebener Verteilung seine optimale Exportmenge reduziert, wenn seine absolute Risikoaversion steigt (vgl. Gollier 2001, S. 59, Prop. 7).

Eine sorgfältige Informationsbeschaffung und -analyse ist wichtig, da die zugrunde gelegte Wahrscheinlichkeitsverteilung einen gravierenden Einfluss auf die optimale Exportmenge ausübt. Ändert sich diese Verteilung, so gelten einige intuitive Vermutungen keineswegs generell. Z. B. verbessere sich die Wahrscheinlichkeitsverteilung des Wechselkurses gemäß stochastischer Dominanz 1. Grades oder 2. Grades. Bei Verbesserung gemäß stochastischer Dominanz 1. Grades wächst definitionsgemäß der erwartete Nutzen des Entscheidungsträgers, unabhängig davon, ob er das Risiko scheut oder liebt. Bei stochastischer Dominanz 2. Grades gilt dies nur für jeden risikoscheuen Entscheider. Man vermutet daher, dass der Exporteur seine optimale Exportmenge erhöht, wenn sich die Wahrscheinlichkeitsverteilung des Wechselkurses gemäß stochastischer Dominanz 1. oder 2. Grades verbessert. Diese Vermutung trifft indessen nicht generell zu. Hadar und Seo (1990) haben gezeigt, dass das optimale riskante Engagement bei einer Verbesserung gemäß stochastischer Dominanz 1. Grades generell nur dann wächst, wenn die relative Risikoaversion des Entscheidungsträgers kleiner als 1 ist, wenn er also wenig risikoscheu ist. Noch schärfere Voraussetzungen gelten bei einer Verbesserung gemäß stochastischer Dominanz 2. Grades, da die Menge der möglichen Verteilungsverbesserungen größer ist (siehe auch Gollier 2001, S. 60, Prop. 9).

Diese Ergebnisse sollten eine Mahnung für die Manager finanzieller und nicht-finanzieller Risiken sein, sich nicht blindlings durch Intuition leiten zu lassen. Bei Risiken mit wenig Information kann neue Information diverse Verteilungsänderungen und damit fragwürdige Entscheidungsänderungen suggerieren.

b) *Export- und Hedgingentscheidung*

Jetzt wird das Entscheidungsmodell um Hedging ergänzt. Besteht ein Terminkontrakt auf den Wechselkurs, so optimiert der Exporteur im Zeitpunkt 0 seine Exportmenge x und die im Terminmarkt verkaufte Menge an Fremdwährung y.^13^ Ist f der Terminkurs im Zeitpunkt 0, dann ergibt sich als Cashflow C(x,y) im Zeitpunkt 1

1 $$\begin{aligned} \mathrm{C}\left(\mathrm{x},\mathrm{y}\right) & =-K_{f}+(\mathrm{p}(\mathrm{x})\mathrm{w}-\mathrm{k}(\mathrm{x}))\mathrm{x}-(\mathrm{w}-\mathrm{f})\mathrm{y}\\ & =-K_{f}+(\mathrm{p}(\mathrm{x})\ \mathrm{f}\ -\mathrm{k}(\mathrm{x}))\mathrm{x}+(\mathrm{p}(\mathrm{x})\mathrm{x}-\mathrm{y})(\mathrm{w}-\mathrm{f})\\ & =-K_{f}+(\mathrm{p}(\mathrm{x})\ \mathrm{f}\ -\mathrm{k}(\mathrm{x}))\mathrm{x}-y_{N}(\mathrm{w}-\mathrm{f}) \end{aligned}$$

$$\mathrm{mit}\ y_{N}=\colon \mathrm{y}-\mathrm{p}(\mathrm{x})\mathrm{x}=\text{Nettoverkauf von Fremdw\"{a}hrung}.$$

Die letzte Zeile der Gleichung zeigt: Das Entscheidungsproblem lässt sich auch anders begreifen. Der Exporteur verkauft seinen Exporterlös zum festen Terminkurs im Terminmarkt, so dass sein Exporterlös in Heimatwährung deterministisch ist. Zusätzlich kann er im Wechselkurs mit dem Verkauf von y_N_ Einheiten spekulieren. Der Exporteur wählt jetzt seine optimale Exportmenge x* so, dass seine Grenzkosten mit dem Grenzerlös, umgerechnet zum Terminkurs in Heimatwährung, übereinstimmen, k(x*) + k′(x*) x* = [*p*(x*) + p′(x*) x*] f. Dieser Sachverhalt wird als Fisher Separation bezeichnet.

Aufschlussreich ist die Änderung in den Informationsanforderungen, die durch den Terminkontrakt entsteht. Für die Exportentscheidung muss der Exporteur lediglich den Terminkurs kennen, nicht aber die Wahrscheinlichkeitsverteilung des Wechselkurses. Die operative Entscheidung wird dadurch sehr vereinfacht (Broll und Wahl 1993). Da die Fisher Separation aus Arbitrage-Überlegungen folgt, bleibt sie gültig, wenn die Information über die Verteilung des Wechselkurses dürftig ist.

Für den Exporteur bleibt die Frage, wie viel Wechselkursrisiko er mit y_N_ nehmen will. Er kann dieses leicht vermeiden, indem er einen full hedge y_N_ = 0 wählt: Er verkauft insgesamt genau den stochastischen Exporterlös im Terminmarkt. Dies ist optimal, wenn der Terminkurs mit dem erwarteten Kassakurs übereinstimmt, d. h. die Wechselkurs-Risikoprämie gleich 0 ist. Wiederum ist eine Information über die Wahrscheinlichkeitsverteilung des Wechselkurses überflüssig, allerdings benötigt der Exporteur eine glaubwürdige Information über die Höhe der Risikoprämie.

Ist die Risikoprämie ungleich 0, so ist eine spekulative Position y_N_ ≠ 0 im Wechselkurs optimal. Hedging- und Spekulationsentscheidung sind zwei Seiten derselben Medaille. Während die Exportentscheidung den erwarteten Nutzen des Exporteurs (und den Marktwert des Unternehmens) erheblich beeinflusst, ändert die spekulative Position im Wechselkurs zwar auch den erwarteten Nutzen, kaum aber den Marktwert des Unternehmens, da ihr Marktwert abgesehen von Transaktionskosten gleich 0 ist. Das Spekulationsergebnis beläuft sich auf die spekulative Position y_N_, multipliziert mit der Differenz von Terminkurs im Zeitpunkt 0 und Kassakurs im Zeitpunkt 1. Das Risiko daraus ist im Allgemeinen erheblich kleiner als das aus dem ungehedgten Exporterlös *p* w x*.

Dieser Sachverhalt eröffnet dem Exporteur unterschiedliche Verhaltensweisen hinsichtlich Informationsbeschaffung, Risikoübernahme und Gestaltung der Organisation. Bei einem full hedge sind die organisatorischen Erfordernisse bescheiden, weil das Wechselkursrisiko keine Rolle spielt und die optimale Exportmenge leicht zu errechnen ist.

Ist es für den Exporteur billig, sich verlässlich über die Wahrscheinlichkeitsverteilung des Wechselkurses zu informieren, dann kann er dies tun und seine spekulative Position im Wechselkurs optimieren. Allerdings muss er organisatorische Vorkehrungen treffen, um die Entscheidung sorgfältig vorzubereiten und später das Risiko zu überwachen. Sind Informationsbeschaffung und Organisation allerdings teuer, dann kann er darauf weitgehend verzichten und seine offene Position im Wechselkurs stark einschränken, ohne seine operative Politik zu ändern. Selbst wenn er sich aus dem Wechselkursrisiko vollständig durch einen full hedge zurückzieht, ist der ihm dadurch entgehende erwartete Nutzen eher gering. Daher lohnt sich für den Exporteur eine spekulative Position im Wechselkurs nur bei geringen Informations- und Organisationskosten. Folglich wird er im Vergleich zur Exportentscheidung ohne Hedging weniger Information beschaffen, wenn es lediglich um die Spekulationsentscheidung geht, Das stützt These 2.

Zahlreiche deutsche Exporteure haben sich nach den Währungsturbulenzen in den 80er Jahren aus der Wechselkursspekulation zurückgezogen, weil sie diese nicht als ihr Kerngeschäft betrachten und der Devisenmarkt außerordentlich kompetitiv geworden ist. Dementsprechend werden Personal- und andere Organisationskosten eingespart.

Aufgrund der Fisher Separation ist die Produktionsentscheidung in diesem Beispiel einfach, so dass in diesem Beispiel Simultan- und Sukzessivplanung ähnlich sind, damit auch die Informationserfordernisse, abweichend von Folgethese 1.

c) *Entscheidung* *bei Wechselkursrisiko und Absatzrisiko*

Das Entscheidungsproblem des Exporteurs wird erheblich komplizierter, wenn er sich auch einem Absatzerlösrisiko und ggf. noch weiteren Risiken gegenübersieht (Adam-Müller 1995, Kap. 3). Das Erlösrisiko lässt sich im Allgemeinen nicht finanziell hedgen, da keine Kontrakte darauf gehandelt werden. Dann gilt die Fisher Separation nicht mehr. Im Newsboy Problem (Eeckhoudt et al. 1995) produziert der Exporteur die Menge x, kann aber damit gegebenenfalls die tatsächliche Nachfrage X nicht decken oder er bleibt auf einem Teil der Produktion sitzen. Im ersten Fall entgehen ihm Gewinne aus entgangener Nachfrage (X − x), im zweiten Fall entgehen ihm Erlöse aus nicht verkauften Einheiten (x − X), abgesehen von möglichen Entsorgungskosten^14^.

Die Stochastik der Nachfrage und ggf. andere Risiken „stochastifizieren“ das Exportergebnis. So kann im Beispiel bei gegebenem Wechselkurs die Höhe des (bedingten) Cashflows C(x,y|w) einem additiven Störfaktor 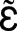 und/oder einem multiplikativen Störfaktor 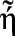 unterworfen werden
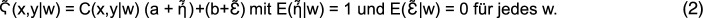


Der bedingte Erwartungswert des Cashflows 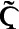 würde durch die Störfaktoren nicht verändert, wenn a = b = 0 wäre. Diese Störfaktoren erzeugen sog. „Hintergrundrisiken“. Wenn die Nutzenfunktion die plausible Eigenschaft abnehmender und konvexer absoluter Risikoaversion (standard risk aversion) aufweist, erhöhen additive Störfaktoren mit einem Erwartungswert b ≤ 0 die Risikoaversion des Entscheidungsträgers (Eeckhoudt et al. 2005, S. 69, Prop. 4.3). Multiplikative Störfaktoren können je nach Nutzenfunktion seine Risikoaversion erhöhen oder senken^15^ (Franke et al. 2006). Bei bestimmten Kombinationen von additivem und multiplikativem Hintergrundrisiko kann die Risikoaversion auch gleich bleiben (Franke et al. 2018).

In Anhang A wird gezeigt, wie bei umfassender Information das Absatzmengenrisiko als multiplikatives Hintergrundrisiko modelliert werden kann. Auch wenn die Unverkäuflichkeit von Produkten in schlechten Absatzzuständen die optimale Produktionsmenge senkt, so wirft die gemeinsame Optimierung von Produktionsmenge und Wechselkursspekulation mit Terminkontrakten offene Forschungsfragen auf. Die Optimierung wird noch komplizierter, wenn es neben linearen auch nicht-lineare Absicherungsinstrumente wie Optionen gibt (Brown und Toft 2002).

d) Entscheidung über Liquiditäts- und Kapitalreserve

Welche Schlussfolgerungen ergeben sich für die Wahl von Liquiditäts- und Kapitalreserve? Das Eigenkapital im Zeitpunkt 1 ergibt sich aus dem im Zeitpunkt 0, korrigiert um Gewinn/Verlust abzüglich Gewinnausschüttungen des Unternehmens und Kapitalzuführungen/entnahmen. Die Liquidität des Unternehmens im Zeitpunkt 1, die z. B. am Geldvermögen des Unternehmens gemessen werden kann, ergibt sich aus der im Zeitpunkt 0, korrigiert um den Cashflow des Unternehmens und monetären Kapitalzuführungen/entnahmen abzüglich Gewinnausschüttungen. Die Höhe der Liquiditätsreserve bemisst sich nach der Wahrscheinlichkeitsverteilung des Geldvermögens im Zeitpunkt 1, die Kapitalreserve nach der des Eigenkapitals im Zeitpunkt 1.

Ausgehend von der Wahrscheinlichkeitsverteilung des Geldvermögens/Eigenkapitals im Zeitpunkt 1 kann eine Reserve nach dem Value at Risk, also einem Quantil der Wahrscheinlichkeitsverteilung, festgelegt werden. Kommt es bei Unterschreiten dieses Quantils zur Insolvenz, so treffen die zusätzlichen Kosten weniger den Exporteur, vielmehr Dritte. Daher mag der Aufseher das Reserveerfordernis nach dem Expected Shortfall bemessen, also nach der Höhe der erwarteten Kosten, die über die dem Expected Shortfall zugrundeliegende Schwelle von Geldvermögen/Eigenkapital hinaus anfallen und Dritte treffen.

Hedgt der Exporteur die Exportrisiken nicht, dann schlagen sie voll auf das zukünftige Geldvermögen/Eigenkapital durch. Die Wahrscheinlichkeit des Ergebnisses zeigt dann einen ausgeprägten negativen Tail. Je mehr der Exporteur seine Risiken hedgt und je weniger er im Wechselkurs spekuliert, umso geringer sind Liquiditäts- und Kapitalreserve. Und umso weniger bedeutsam und damit weniger lohnend ist die Schätzung des negativen Tails für die Reservebildung.

Da es bei der Bemessung von Reserven nur auf den negativen Tail ankommt, sind insoweit die Informationsbeschaffung und organisatorischen Erfordernisse weniger aufwändig als bei Entscheidungen, bei denen es auf die gesamte Wahrscheinlichkeitsverteilung des Ergebnisses ankommt.

### Entscheidung bei eingeschränkter Information

Entscheidungsregeln können an die Qualität der verfügbaren entscheidungsrelevanten Information gekoppelt werden. Bisher wurde umfassende Information unterstellt. Wie reagiert der Entscheidungsträger auf eine Verschlechterung der Informationsqualität? Die Modelle, die in der Literatur vorgeschlagen werden, bieten möglicherweise eine Orientierung für den Umgang mit nicht-finanziellen Risiken. Vier Modelle zum Vorgehen bei geringer Informationsqualität werden im Folgenden skizziert. Um diese Modelle zu erläutern, wird von einer üblichen Ergebnismatrix mit endlich vielen Zuständen und Handlungsalternativen ausgegangen, die für jeden Zustand der Natur und für jede Handlungsalternative das zugehörige Ergebnis zeigt. Bei umfassender Information sind die Wahrscheinlichkeiten der Zustände bekannt. Eine Verschlechterung der Informationsqualität kann den Entscheidungsträger veranlassen, bei unveränderter Ergebnismatrix und unveränderten Wahrscheinlichkeiten seine Risikoneigung der Qualität anzupassen (Pauschalmodell). Oder er passt die Nutzenfunktion des Ergebnisses in den einzelnen Zuständen an die Qualität an (Hintergrundrisiko-Modell) oder er passt die Zustandswahrscheinlichkeiten an (Ambiguitätsmodell) oder er unterstellt lediglich qualitative Wahrscheinlichkeiten. Welche Vorgehensweise unter welchen Bedingungen am sinnvollsten ist, bleibt zu klären.

1. *Pauschalmodell*: Eine einfache pauschalierte Reaktion auf eine Verschlechterung der Informationsqualität kann darin bestehen, die Risikoaversion der verwendeten Nutzenfunktion pauschal zu erhöhen, also den Risikoappetit zu senken. Der Entscheidungsträger nimmt dann ein insgesamt geringeres Risiko, wenn er trotz Verschlechterung der Information von denselben Wahrscheinlichkeitsverteilungen der Ergebnisse ausgeht. Analog kann die Höhe von Liquiditäts- und Kapitalreserve an die Informationsqualität so gekoppelt werden, dass eine Verschlechterung der Informationsqualität zu einer pauschalen Erhöhung der Reserven führt. So kann der Entscheidungsträger bei schlechterer Information das Quantil verschärfen (z. B. von 1 auf 0,5 %) bzw. die dem Expected Shortall zugrundeliegende Schwelle erhöhen. Dann wachsen die Reserveerfordernisse. Dieses Verhalten bildet die normative These 5 ab.

#### These 5:

*Der auf Informationsqualität bedachte Entscheidungsträger reagiert auf eine Verschlechterung der Qualität in seinen Entscheidungsmodellen mit einer pauschalen Erhöhung seiner Risikoaversion und mit einer pauschalen Erhöhung der Liquiditäts- und Kapitalreserven*.

2. *Modell mit Hintergrundrisiken*: Die Verschlechterung der Informationsqualität kann auch über die Einführung von Hintergrundrisiken modelliert werden, die bereits in Gleichung (2) eingeführt wurden. Ein Beispiel für ein additives Hintergrundrisiko wäre eine unklare Information über die Höhe der fixen Produktionskosten, so dass diese zu (K_f_ +  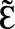) randomisiert werden. Beispiele für ein multiplikatives Hintergrundrisiko wären Absatzerlösrisiken diverser Art oder auch eine unklare Information über die Höhe der Inflation, wenn der Cashflow C in nominalen Einheiten gemessen wird, jedoch der Nutzen von realen Geldeinheiten abhängt. Die nominalen Geldbeträge sind dann mit einem stochastischen Kaufkraftindex 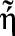 zu multiplizieren.

Bei umfassender Information gilt 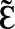
 = 0 und 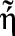
 = 1. Je geringer die Informationsqualität ist, desto mehr streuen 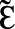 und 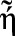, gemessen z. B. an ihrer Standardabweichung. Bildet man im Beispiel bei gegebenem Wechselkurs den Erwartungswert der ursprünglichen Nutzenfunktion, E[U(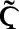
(x,y|w))], über die Störvariablen 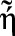 und 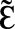, so definiert dieser die indirekte Nutzenfunktion U*[C(x,y|w)]. Die optimale Produktionsmenge und das optimale Hedgevolumen werden sodann errechnet, indem der Erwartungswert der indirekten Nutzenfunktion über den Wechselkurs gebildet und maximiert wird. Die Abnahme der Informationsqualität führt in diesem Modell dazu, dass in jedem Zustand der Nutzen U[C(x,y|w)] durch den Nutzen U*[C(x,y|w)] ersetzt wird.

Wie bereits ausgeführt, hängt es von der ursprünglichen Nutzenfunktion ab, ob die Risikoaversion der indirekten Nutzenfunktion größer oder kleiner ist als die der ursprünglichen. So ist es möglich, dass die Hintergrundrisiken die Risikoaversion des Entscheidungsträgers senken und damit Alternativen mit Hintergrundrisiko attraktiver machen. Dieser Effekt kann mit der Höhe der Hintergrundrisiken wachsen, also dann, wenn die Informationsqualität abnimmt. Bei anderen Nutzenfunktionen ist dies umgekehrt.

Entgegen These 5 ist offen, ob der Entscheidungsträger bei schlechterer Informationsqualität höhere Reserven wählt, wenn seine Risikoaversion abnimmt. Denn die Reserven bemessen sich nach dem wahrgenommenen Risiko, das eher wächst, und der Risikoaversion.

3. *Ambiguitätsmodell*: Eine Verschlechterung der Informationsqualität kann auch über ein Ambiguitätsmodell erfasst werden. Bei Ambiguität gibt es keine eindeutige Wahrscheinlichkeitsverteilung des Ergebnisses. Eine Möglichkeit, Ambiguität zu modellieren, geht davon aus, dass es für eine Ergebnisvariable mehrere Wahrscheinlichkeitsverteilungen gibt, die ihrerseits selbst mit bestimmten Lotterie-Wahrscheinlichkeiten eintreten^16^. Gilboa und Schmeidler (1989) haben axiomatisch begründet, dass ein ambiguitätsaverser Entscheider für eine gegebene Handlungsalternative aus den zu Grunde liegenden Verteilungen diejenige heraussucht, bei der ihr erwarteter Nutzen minimal ist. Er wählt dann die Handlungsalternative, bei der dieser minimale erwartete Nutzen maximal ist. Analog könnte er bei gegebener Handlungsalternative diejenige Wahrscheinlichkeitsverteilung des Ergebnisses, die zu den höchsten Liquiditäts- und Kapitalreserven führt, der Reservebildung zugrunde legen. Das entspricht einer sehr pessimistischen Sichtweise.

Um verschiedene Grade der Ambiguitätsaversion abbilden zu können, wurden verschiedene Modelle entwickelt. Es liegt nahe, für eine gegebene Entscheidungsalternative den Erwartungsnutzen des Ergebnisses für jede mögliche Wahrscheinlichkeitsverteilung zu berechnen und dann die verschiedenen Erwartungsnutzen mit den Lotteriewahrscheinlichkeiten zu multiplizieren und zu einem Gesamtnutzen zu aggregieren. Nach dem von Neumann Morgenstern-Prinzip spielt Ambiguität indessen keine Rolle, weil nach dem Unabhängigkeitsaxiom die Wahrscheinlichkeiten eines Zustands in den verschiedenen Verteilungen mit den Lotterie-Wahrscheinlichkeiten multipliziert und zu einer erwarteten Zustandswahrscheinlichkeit addiert werden.

Eine jüngere Theorie wurde von Izhakian (2020)^17^ (weiter)entwickelt. Danach kann der ambiguitätsaverse Entscheidungsträger seinem Entscheidungskalkül die erwartete oder mittlere Wahrscheinlichkeit eines Zustands, ergänzt um einen Ambiguitätsabschlag/zuschlag, zugrunde legen. Der Abschlag/Zuschlag wächst mit der Standardabweichung der möglichen Wahrscheinlichkeiten des Zustands um ihren gegebenen Erwartungswert und der Höhe der Ambiguitätsaversion des Entscheidungsträgers.

In Anlehnung an Kahnemann und Tversky geht Izhakian von einem Referenzpunkt des Ergebnisses aus. Ergebnisse oberhalb dieses Referenzpunktes gelten als wünschenswert, Ergebnisse unterhalb als unerwünscht. Der Entscheidungsträger kann in Reaktion auf Ambiguität die mittlere Wahrscheinlichkeit eines erwünschten Zustands um einen Abschlag vermindern und die mittlere Wahrscheinlichkeit eines unerwünschten Zustands um einen Zuschlag erhöhen. Die Summe der korrigierten Wahrscheinlichkeiten kann unter 1 liegen. Der mit diesen korrigierten Wahrscheinlichkeiten errechnete Erwartungsnutzen einer Handlungsalternative ist dann umso kleiner, je größer deren Ambiguität ist. Alternativen mit höherer Ambiguität werden mehr „abgestraft“ und verlieren dadurch an Attraktivität.

Wendet man dieses Konzept auch auf die Reservebildung an, so werden die Wahrscheinlichkeiten der Zustände mit schlechten Ergebnissen erhöht. Der Value at Risk wächst bei unverändertem Quantil, ebenso auch der Expected Shortfall bei gegebener Shortfall-Schwelle.

4. *Modell mit qualitativen Wahrscheinlichkeiten*: Menschen sind eher in der Lage, für die Wahrscheinlichkeit eines Zustands ein Intervall anzugeben, in dem sie liegt, anstelle einer Zahl, also eines Intervalls mit der Breite 0. Noch leichter mag es sein, qualitative Wahrscheinlichkeiten anzugeben. Ausgangspunkt kann der einzelne Zustand sein, aber auch ein Ereignis, das als Teilmenge von Zuständen definiert ist. Diese Ereignisse seien disjunkt, d. h. jeder Zustand ist als Element in genau einem Ereignis enthalten. Qualitative Wahrscheinlichkeiten existieren vollständig, wenn der Entscheidungsträger in der Lage ist, für jedes Paar von Ereignissen anzugeben, welches Ereignis wahrscheinlicher ist. Ist diese Voraussetzung erfüllt, dann lässt sich die Menge der Ereignisse nach den qualitativen Wahrscheinlichkeiten sortieren. Je feiner die Ereignisse definiert sind, d. h. je kleiner die zu einem Ereignis gehörende Teilmenge von Zuständen ist, umso anspruchsvoller ist das Erfordernis einer vollständigen Angabe qualitativer Wahrscheinlichkeiten.

Zu qualitativen Wahrscheinlichkeiten existieren jeweils konsistente quantitative Wahrscheinlichkeiten. Wie in Anhang B verdeutlicht wird, bestehen auch bei qualitativen Wahrscheinlichkeiten für jedes Ereignis eine minimale und eine maximale quantitative Wahrscheinlichkeit und damit ein Intervall. Das Intervall ist größer für ein Ereignis a als für ein Ereignis b, wenn Ereignis a qualitativ wahrscheinlicher ist; es wächst auch, wenn die Zahl der anderen, vordefinierten Ereignisse abnimmt.

Ausgehend von Axiomen untersucht Bühler (1975, 1981) dieses Modell. Im Ergebnis kommt er wie Gilboa und Schmeidler (1989) zu einer pessimistischen maxmin- Entscheidungsregel.

Welche der skizzierten Vorgehensweisen unter welchen Bedingungen am sinnvollsten ist, bleibt offen. Dazu bedarf es weiterer Forschung. Unabhängig davon lohnt sich bei schlechterer Information eine umfangreichere Informationsbeschaffung, sofern es dadurch gelingt, die Qualität der Information und damit die der Entscheidung zu verbessern.

### Minimale Informationsanforderungen?

- Willkür bei der Vorgabe der Ergebnismatrix?

Die bisherigen Ausführungen unterstellen, dass der Entscheidungsträger auch bei schlechter Informationsqualität die Ergebnismatrix kennt und Wahrscheinlichkeitsverteilungen in zumindest grober Form schätzen kann. Eine wichtige Forschungsfrage lautet, welche minimalen Anforderungen an die Informationsqualität zu stellen sind, damit die Entscheidung besser fundiert wird als bei Kaffeesatzlesen.

Entscheidungsgrundlage ist eine sorgfältig erstellte Ergebnismatrix. Sie ist selbst das Ergebnis vorgelagerter Entscheidungen des Entscheidungsträgers. Diese sollten die optimale Handlungsalternative nicht präjudizieren, sondern eine unverzerrte Basis für deren Wahl bereitstellen. Zwei Fragen werden hier aufgegriffen. 1) Welche Zustände werden in der Ergebnismatrix berücksichtigt, welche nicht? 2) Wie fein werden Zustände/Ereignisse differenziert?

Ad 1): In der Ergebnismatrix werden nur Zustände berücksichtigt, deren Eintritt als möglich erachtet wird. Auch hierbei gibt es jedoch Fühlbarkeitsschwellen. Zustände, die nur extrem selten beobachtet werden, werden häufig ausgeklammert^18^. In diesen Zuständen können hohe oder niedrige Ergebnisse mit entsprechend hohen oder niedrigen Ergebnisnutzen auftreten. Solange es verlässliche Wahrscheinlichkeiten gibt, spielen extrem selten auftretende Zustände für den Erwartungsnutzen einer Handlungsalternative indessen eine geringe Rolle, da ihre Eintrittswahrscheinlichkeit gegen 0 tendiert. Daher ist der Einfluss dieser Zustände auf die Optimierung gering, folglich auch der Einfluss der vorgelagerten Entscheidung, Zustände in die Ergebnismatrix aufzunehmen oder nicht.

Anders ist es, wenn keine Wahrscheinlichkeiten bekannt sind. Dann sind alle Zustände in der Ergebnismatrix „gleichrangig“. Dies verschafft den extrem selten auftretenden Zuständen erhebliches Gewicht. Dies verdeutlicht die Anwendung klassischer Entscheidungskriterien bei Unsicherheit (Friberg 2015, Ch. 9). So ist gemäß der Maxmin-Regel die Alternative zu wählen, deren schlechtestes Ergebnis am höchsten ist. Dies bedeutet, dass Alternativen mit geringer Schwankungsbreite des Ergebnisses in die engere Wahl kommen und solche mit großer Schwankungsbreite (= hochriskante Alternativen) eher verdrängt werden. Dieser Verdrängungseffekt ist umso stärker, je mehr selten auftretende Zustände mit sehr niedrigen Ergebnissen hochriskanter Alternativen berücksichtigt werden. Damit präjudiziert die Vorgabe der Ergebnismatrix, inwieweit wenig riskante Alternativen hoch riskante verdrängen. Dies macht wenig Sinn.

Gemäß der Hurwicz-Regel wird für jede Alternative ein gewogenes Mittel von schlechtestem und bestem Ergebnis ermittelt und die Alternative mit dem höchsten Mittel gewählt. Auch wenn diese Regel den Verdrängungseffekt einschränkt, bleibt unbefriedigend, dass lediglich extreme Ergebnisse, die eventuell extrem selten auftreten, die Entscheidung determinieren. Wiederum erweist sich als maßgeblich, welche Zustände in die Matrix einbezogen werden. Soweit diese Wahl wenig begründet ist, trifft dies auch für die optimale Alternative zu.

Ad 2): Die Vorgabe einer Ergebnismatrix setzt weitere mehr oder weniger willkürliche Vorab-Entscheidungen voraus, so über die Zahl der zu berücksichtigenden Risikofaktoren, über die Vorgabe eines Zahlenintervalls, in dem die Realisation eines Risikofaktors liegen kann, und über die Feinheit der Aufteilung dieses Intervalls.^19^ Oft werden die Ergebnisse von einer großen Zahl von Risikofaktoren getrieben. Selbst wenn deren Definitionsbereich eingegrenzt werden kann, explodiert die Zahl der Zustände schnell. Gibt es *n* Risikofaktoren und jeweils k verschiedene Realisationen, die beliebig kombiniert werden können, dann gibt es k^n^ Zustände. Die Problematik der Zustandsauswahl wird deutlich, wenn der Entscheidungsträger ein (gewogenes) Mittel der Ergebnisse über alle Zustände errechnet und danach entscheidet. Auch hierbei erweist sich die Zustandsauswahl als entscheidend. Denn sie determiniert das Mittel der Ergebnisse und damit die Rangordnung der Handlungsalternativen.

Sollen die Vorab-Entscheidungen über die Ergebnismatrix nicht willkürlich sein, so erscheinen (grobe) Wahrscheinlichkeitsurteile unverzichtbar, die ihrerseits möglichst evidenzfundiert sein sollten. Auch sollten diese Entscheidungen anhand einer Kosten-Nutzen-Analyse getroffen werden. Hat z. B. ein nicht-finanzielles Risiko oder ein Risikofaktor vermutlich nur einen geringen Einfluss auf die Ergebnisse der Handlungsalternativen, dann mag es in Anbetracht der Informations- und Transaktionskosten des Entscheidungs- und Umsetzungsprozesses sinnvoll sein, dieses Risiko bzw. diesen Risikofaktor zu vernachlässigen.

b) Willkür bei der Szenarien-Analyse?

Die Problematik einer Festlegung der Ergebnismatrix trifft auch die weit verbreitete Szenario-Technik^20^. Um bei Fehlen von Wahrscheinlichkeiten einen Einblick in Ertrag und Risiko von Handlungsalternativen zu gewinnen, werden Ereignisse zu „repräsentativen Szenarien“ aggregiert, die das Spektrum der möglichen Zustände abbilden sollen, z. B. Szenarien mit sehr schlechten, mit mittleren und mit sehr guten Ergebnissen. Die Zahl der aus einer Teilmenge von Zuständen ausgewählten Szenarien sollte mit dem „Repräsentationsgewicht“ dieser Teilmenge wachsen. So kann versucht werden, Ergebnisse operativer Handlungsalternativen grob zu kennzeichnen und auf dieser „Evidenzgrundlage“ zu entscheiden. Das Fehlen von Wahrscheinlichkeiten wird durch Plausibilitätskonstrukte „geheilt“, die indessen vage bleiben. Gibt es bessere Vorgehensweisen?

Die Problematik kann am Maximum Probable Loss veranschaulicht werden, der in der Versicherungswirtschaft eine wichtige Rolle spielt. Wenn ein Versicherer einen Kunden gegen Cyber-Risiken versichern möchte, kann er für seine eigene Kalkulation versuchen, den Maximum Probable Loss des Kunden abzuschätzen, der konzeptionell dem Value at Risk verwandt ist. Bisher gibt es zu möglichen Cyber-Schäden und deren Häufigkeiten nur wenig verlässliche Information. Dieser Informationsmangel wird auch beim „Maximum Probable Loss“ sichtbar. Der Begriff ist in sich widersprüchlich, da zwar ein „worst-case scenario“ des Schadens gemeint ist, jedoch eine Art „realistic worst case“, also ein „worst case“ mit nicht vernachlässigbarer Eintrittswahrscheinlichkeit. Somit wird nicht das schlechtestmögliche Ergebnis der Ergebnismatrix verwendet, sondern ein Pseudoquantil, dessen Ermittlung allerdings in Anbetracht der schlechten Informationsqualität auf fragwürdigen Grundlagen beruht. Daher ist der Maximum Probable Loss von subjektiven Einschätzungen geprägt und bleibt eine vage Größe, die von unterschiedlichen Versicherern unterschiedlich eingeschätzt wird.

Ein zweites Beispiel liefert der Expected Shortfall. Zur Abschätzung nutzen Regulatoren/Aufseher mehrere bad case-Szenarien, anhand derer sie über eine vorgegebene Schwelle hinausgehende Verluste berechnen und zu einem Expected Shortfall aggregieren. Das Ergebnis hängt naturgemäß stark von den zugrunde gelegten bad case-Szenarien und deren Gewichtung ab.

Dieser Sachverhalt eröffnet ein Feld für Manipulationen und erfordert daher organisatorische Vorkehrungen. Wer soll z. B. in die Festlegung von Szenarien für ein Entscheidungsproblem eingebunden sein? Einerseits können dazu Personen gehören, die dank ihrer Tätigkeit mit dem Entscheidungsproblem besonders gut vertraut sind, ebenso übergeordnete Entscheidungsträger, andererseits aber auch Personen, die kein „skin in the game“ haben und daher einen neutralen Blick auf mögliche Szenarien werfen können. Das ähnelt einem 3 lines of defense-Modell.

Die Anwendung der Szenarien-Technik ist weniger problematisch, wenn es um spezifisches Hedging geht und es preiswerte Hedging-Instrumente gibt, um Ergebnisschwankungen weitgehend auszugleichen. Das gilt insbesondere, wenn es sich um finanzielle Hedging-Instrumente mit einem Marktwert nahe 0 handelt.^21^ Dann spielt die Auswahl von Szenarien für die Hedgingentscheidung eine untergeordnete Rolle. Diese Überlegungen knüpfen an These 2 an.

Je geringer die Hedgequalität ist, umso größer ist das Basisrisiko, gemessen an den möglichen Abweichungen zwischen dem zu hedgenden operativen Ergebnis und dem Hedge-Ergebnis. Dann gewinnt die Auswahl der Szenarien eine größere Bedeutung. Diese Überlegungen motivieren Folgethese 2 zu These 2.

#### Folgethese 2:

*Bei schlechter Informationslage ist die Auswahl der Szenarien für die Hedgingentscheidung umso wichtiger, je schlechter die erzielbare Hedge-Qualität ist*.

Die schwierige Problematik, der sich Entscheidungsträger einschließlich Regulatoren und Aufseher bei bescheidener Information gegenübersehen, schließt verlässliche evidenzbasierte Entscheidungen weitgehend aus. Daher gewinnt die Beschaffung weiterer Informationen hohes Gewicht. Dies veranschaulicht auch das Vorgehen der Versicherer bei Nutzung des Maximum Probable Loss. Vor Abschluss einer Versicherung versuchen sie, ihre verfügbare Information deutlich zu verbessern. Dabei helfen einerseits weltweite Erhebungen zu Cyber-Angriffen und dadurch ausgelösten Schäden, andererseits intensive Verhandlungen über Vorsichtsmaßnahmen mit dem Kunden im Vorfeld des Vertragsabschlusses. Diese dienen dazu, die Wahrscheinlichkeit eines Cyber-Angriffs zu vermindern^22^ und über geeignete Notfallpläne die Höhe des Schadens nach Eintritt eines Schadensfalls zu vermindern. Es wird also versucht, durch ein vorab zu vereinbarendes Maßnahmenpaket potentielle Schäden zu senken und Unsicherheit abzubauen. Dies deutet auf einen generellen Zusammenhang, den These 6 formuliert.

#### These 6:

*Je schlechter die verfügbare Information ist, desto attraktiver werden im Vorfeld einer Entscheidung festzulegende ex-ante Vorsichtsmaßnahmen und ex post-Notfallpläne, um die möglichen Entscheidungsergebnisse und gleichzeitig die verfügbare Information zu verbessern*.

Diese These gilt ebenfalls ceteris paribus, also bei ansonsten gegebener Informationsbeschaffungsstrategie, kurz: Informationsstrategie. Sie bezeichnet die Vorgehensweise des Entscheidungsträgers, um seinen Informationsstand nicht nur im Zeitablauf zu aktualisieren, sondern ggf. auch darüber hinaus zu verbessern. Im nächsten Abschnitt wird sie eingehend erörtert. Sie variiert mit der Qualität der verfügbaren Information. So mag es bei jungen Start-ups wenig Sinn machen, Cashflows zu prognostizieren. Existiert das Unternehmen bereits einige Jahre, dann erlaubt die gewonnene Erfahrung, Cashflows verlässlicher zu prognostizieren, so dass diese Prognose Teil der Informationsstrategie wird.

Die vorangehenden Erwägungen legen nahe, dass es ohne grobe subjektive Wahrscheinlichkeiten nicht möglich ist, Entscheidungen sinnvoll zu treffen. Das wird noch deutlicher bei Handlungsalternativen, deren Ergebnisverteilung zur mathematischen Bequemlichkeit auf dem Intervall (−∞,+∞) definiert wird. Dann sind extreme Ergebnisse nicht mehr definiert. Wenn es nicht gelingt, durch grobe subjektive Wahrscheinlichkeitsvorstellungen die potentiellen Ergebnisse näher zu charakterisieren, ist eine gezielte Informationsbeschaffung unerlässlich. Die Beschaffung und Verarbeitung setzen indessen erheblich komplexere Entscheidungs- und Organisationsprozesse voraus. Sie werden im folgenden Abschnitt näher diskutiert, insbesondere die damit verbundenen offenen Fragen.

## Flexible Planung und Unternehmensstrategie bei Informationsdefiziten

### Varianten flexibler Planung

Je weniger Information aktuell verfügbar ist, desto mehr Bedeutung gewinnen Unternehmensstrategien, die einerseits zwar unaufschiebbare Entscheidungen optimieren, andererseits aber prüfen, inwieweit andere Entscheidungen mit Vorteil hinausgeschoben werden können. Der Vorteil des Hinausschiebens von Entscheidungen besteht darin, dass in der Zwischenzeit neue Informationen eintreffen, so dass Entscheidungen dann auf präziser prognostizierbaren Ergebnissen beruhen. Wenn ein Entscheidungsträger eine Entscheidung verzögert, nutzt er eine Realoption des Abwartens, kurz: Warteoption (Chevalier-Roignant und Trigeorgis 2011; Dixit und Pyndick 1994).

Das Hinauszögern einer Entscheidung kann jedoch auch Wartekosten verursachen, z. B. wenn eine Verlustquelle nicht abgestellt wird, oder wenn Entscheidungen, die zu einem bestimmten zukünftigen Zeitpunkt umgesetzt sein müssen, hinausgezögert werden. Wenn z. B. eine Kapitalerhöhung bis zu einem vorgegebenen Zeitpunkt abgeschlossen sein muss, wird sie häufig teurer, wenn sie später beschlossen und daher rascher durchgeführt wird. Potentielle Finanziers haben dann weniger Zeit, sich Informationen über die geplante Maßnahme zu beschaffen. Dafür stellen sie evt. höhere Finanzierungskosten in Rechnung, sie reagieren auf eine Verkürzung ihrer Warteoption mit höheren Finanzierungskosten. Daher empfiehlt sich ein frühzeitiger Aufbau von Liquiditäts- und Kapitalreserven.

Der optimale Entscheidungszeitpunkt verschiebt sich tendenziell umso mehr in die Zukunft, je geringer die Wartekosten pro Zeiteinheit sind und/oder je höher der Wartevorteil pro Zeiteinheit ist. Der Wartevorteil ist tendenziell bei weniger verfügbarer Information größer, weil dann mehr Information pro Zeiteinheit zufließt und somit eine bessere Fundierung von Entscheidungen verstärkt. Dies motiviert die folgende

#### These 7:

*Je weniger Information aktuell verfügbar ist, desto wertvoller ist die Warteoption*.

Modelle flexibler Planung eignen sich, durch Informationsmangel erschwerte Entscheidungsprobleme abzubilden (Friberg 2015, Ch. 8). Im klassischen Modell der flexiblen, zeitdiskreten Planung gibt es die Zeitpunkte 0, 1, 2, …, T (Hespos und Strassmann 1964; Laux 1971). Entscheidungen können in allen Zeitpunkten außer dem letzten getroffen werden. Die Zustandsknoten in diesen Zeitpunkten sind gleichzeitig Entscheidungsknoten. Lediglich die Knoten im Zeitpunkt T (Planungshorizont) sind Knoten, die nur der abschließenden Feststellung von Ergebnissen dienen. Jeder Entscheidungsknoten ist vollständig gekennzeichnet durch die dann vorhandene Information, die auch beobachtbare Ergebnisse früherer Entscheidungen beinhalten kann, die momentan gegebenen Handlungsmöglichkeiten und die bedingten Übergangswahrscheinlichkeiten zu den Folgeknoten. Die optimalen Entscheidungszeitpunkte werden automatisch ausgewählt, sofern die Möglichkeiten, Entscheidungen zu verschiedenen Zeitpunkten zu treffen, im Modell korrekt abgebildet werden. Die in einem Knoten verfügbare Information wird häufig exogen modelliert, d. h., sie wird von exogenen Risikofaktoren getrieben. Diese bestimmen auch die in einem Knoten beobachtbaren Ergebnisse früherer Handlungen. Der Lernprozess des Entscheidungsträgers ist somit exogen vorgegeben.

Der Entscheidungsträger kann jedoch außerdem Informationsbeschaffung über separate Maßnahmen planen und damit seine Informationsstrategie ausbauen und verfeinern. Dies liegt insbesondere bei nicht-finanziellen Risiken infolge der dürftigen Information nahe. Dann wird der exogene Informationsprozess durch einen endogenen ergänzt: Informationsbeschaffungsmaßnahmen werden in das flexible Modell eingefügt. Diese Maßnahmen können z. B. dazu dienen, Information über weitere Risikofaktoren zu sammeln. Deren Bedeutung für das Unternehmen mag auch von zuvor getroffenen Entscheidungen abhängen und daher einem endogenen Prozess unterliegen, so dass auch die Intensität optimaler Informationsbeschaffung diesem Prozess unterliegt. Ebenfalls kann der Entscheidungsträger Informationen beschaffen, um die Qualität der Übergangswahrscheinlichkeiten zu verbessern. Besonders wichtig sind indessen Forschung und Entwicklung, um mit ihren Erkenntnissen neue Geschäftsmodelle zu erschließen.

Es liegt nahe, operative Maßnahmen so zu wählen, dass damit hoffentlich ein operativer Erfolg erzielt wird und gleichzeitig wertvolle Information zufließt. Dies ist typisch für Pilotprojekte. Z. B. wird in einem Testgebiet ein neues Produkt angeboten und damit gleichzeitig Information über die voraussichtliche Akzeptanz des Produktes in anderen Gebieten gewonnen. Ein klassisches Beispiel liefert die internationale sequentielle Expansionsstrategie von Unternehmen. Meist startet ein nationales Unternehmen in nur einem ausländischen Markt mit dem Verkauf seiner Produkte, um deren Attraktivität für Kunden und mögliche Reaktionen von Wettbewerbern zu testen. Hierbei wird nur ein kleinerer Betrag investiert, um das Risiko zu begrenzen. Erweist sich der Vorstoß als erfolgreich, werden die Aktivitäten im diesem Testmarkt ausgebaut und auf weitere Auslandsmärkte ausgedehnt, usw. Das positive Feedback senkt auch das Risiko weiterer Investitionen und erlaubt daher, das Investitionsvolumen zu erhöhen. Gibt es dann bei weiteren Expansionsschritten einen Rückschlag, so werden die internationalen Aktivitäten angepasst und ggf. wieder heruntergefahren.

Mit aktiver Informationsbeschaffung wächst die Menge der Knoten im flexiblen Planungsmodell deutlich. Dagegen kann eingewendet werden, dass von vornherein im Ausgangsmodell alle solchen denkbaren Knoten enthalten sein sollten. Damit würde dieses allerdings enorm aufgebläht, seine Handhabung viel schwieriger und kostspieliger.

### Zur Komplexität von Entscheidungsmodellen

Wie komplex soll das flexible Planungsmodell sein? Auf Grenzen der Komplexität hat bereits Herbert Simon (1957) mit Betonung der begrenzten Rationalität von Individuen hingewiesen. Das Zusammenwirken mehrerer Risikofaktoren auf das Ergebnis einer Entscheidung ist für ein Individuum schon im statischen Modell schwer zu verstehen, geschweige denn in einem stochastisch sequenziellen Modell. Es liegt daher nahe, ein flexibles Planungsmodell zu programmieren und mithilfe eines fortgeschrittenen Rechners Entscheidungen zu ermitteln. Aber auch die Lösung von flexiblen Planungsmodellen mit modernen Computern stößt schnell an Grenzen, wenn es mehr als eine Handvoll von Risikofaktoren gibt.

Selbst wenn der Rechner komplizierte Entscheidungsmodelle lösen könnte, bleibt offen, ob die damit verbundenen Verwaltungskosten gerechtfertigt sind, sofern die dem Modell zu Grunde liegende Information wenig verlässlich ist. Je geringer deren Verlässlichkeit ist, desto weniger verlässlich sind auch die errechneten Entscheidungen. Machen flexible Planungsmodelle auf mittlere oder längere Frist dann noch Sinn?

Diese Skepsis veranlasste Williamson (1975) zu der Schlussfolgerung, dass komplexe Verträge, auch wenn sie Regeln für zahlreiche Umweltentwicklungen vorsehen, wenig effektiv sind. Es kommt zum Marktversagen. An die Stelle einer Kooperation von Individuen über den Markt mithilfe von Verträgen sollte dann die Kooperation innerhalb eines Unternehmens mit geeigneter Organisation treten. Da die Mitarbeiter des Unternehmens sich jederzeit selbst innerhalb des arbeitsvertraglichen Rahmens, der viele Einsatzoptionen umfasst, flexibel koordinieren können, kann das Unternehmen schnell und flexibel reagieren. Allerdings kann es auch zu Organisationsversagen kommen. Gründe hierfür können fragwürdige Anreizsysteme für die Mitarbeiter sein, ebenso Informationsasymmetrien, die zu vielfältig diskutierten Agency Problemen führen. Es gilt daher, eine geeignete Aufbauorganisation mit einer geeigneten Prozessorganisation, die den Ablauf interner Arbeitsprozesse und die Vergabe von Anreizen steuert, zu verbinden. Was geeignet ist, mag bei umfassender Information erkennbar sein. Bei dürftiger Information ist dies indessen unklar. Daher ist infolge häufiger auftretender „Informationsschocks“ auch die Corporate Governance einem rascheren Trial and Error-Prozess unterworfen. Auch dies erklärt, weshalb die Praxis beim Umgang mit nicht-finanziellen Risiken um wissenschaftliche Unterstützung nachsucht.

Eines der ungelösten Probleme: Unter welchen Bedingungen reduziert eine Verschlechterung der verfügbaren Information die gewünschte Komplexität des Entscheidungsmodells und stellt gleichzeitig höhere Anforderungen an die Aufbau- und Prozessorganisation? Besteht eine Substitutionalität zwischen beiden?

Diese Zusammenhänge sollen anhand möglicher Gestaltungsformen der Corporate Governance veranschaulicht werden. Das Entscheidungsmodell kann vereinfacht werden, indem z. B.

- für einen kürzeren Planungszeitraum geplant und dieser revolvierend hinausgeschoben wird,
- die Zahl der Perioden im Modell reduziert wird,
- die Zahl der Knoten in einem Modellzeitpunkt reduziert wird oder
- weniger Entscheidungen oder Entscheidungen vergröbert im Modell abgebildet werden.

Welches Portfolio von Vereinfachungen ist unter welchen Bedingungen sinnvoll, insbes. bei wenig Information? Z. B. wird in Modellen der flexiblen Planung die Länge der Perioden vorgegeben, sie ist jedoch selbst Gegenstand der Optimierung. Einige Effekte einer Periodenverkürzung werden kurz vorgestellt:

- Bei gegebenem Planungshorizont erhöht eine Periodenverkürzung die Zahl der Teilperioden, Information wird häufiger beschafft und ausgewertet. Dann kann die Informationsbeschaffung in jedem Beschaffungszeitpunkt vereinfacht werden. Z. B. gehorcht ein Risikofaktor einem GARCH-Prozess, so dass seine Wahrscheinlichkeitsverteilung mit zunehmendem Zeithorizont breiter wird, ebenfalls das Intervall der nicht vernachlässigbaren Ausprägungen des Risikofaktors. Die Prognose wird damit schwieriger und teurer. Bei flexibler Informationsbeschaffung mit kürzeren Perioden wird das relevante Intervall, in dem der Risikofaktor im nächsten Zeitpunkt liegen kann, verkleinert, ebenso kann das relevante Intervall im nächsten Prognosezeitpunkt gemäß der dann beobachteten Ausprägung des Risikofaktors gezielter angepasst werden. Beides senkt die Informationskosten, andererseits steigen sie infolge häufigerer Beschaffung und Auswertung.
  Zudem erlaubt eine Periodenverkürzung, die Präzision der jeweils zu beschaffenden Information zu reduzieren. Denn Mängel in der Informationsbeschaffung können dann zum früheren Beginn der nächsten Periode rascher korrigiert werden.
- Die Periodenverkürzung erhöht die Zahl der Perioden und damit der Entscheidungszeitpunkte. Es entstehen zusätzliche Realoptionen. Diese beinhalten evt. nicht nur neue operative Maßnahmen, sondern auch die raschere Korrektur früher umgesetzter Maßnahmen, die sich bereits als „Fehlentscheidungen“ herausgestellt haben. So mag es bei geringer Information sinnvoll sein, eine bestimmte operative Maßnahme auszutesten, auch wenn deren Erfolge schlecht abschätzbar sind. Umso wichtiger ist es dann, die erzielten Erfolge kurzfristig zu beobachten und die Vorgehensweise dementsprechend anzupassen. Je weniger Information verfügbar ist, umso mehr Gewicht gewinnt die Reversibilität von Maßnahmen. Diese ist umso geringer, je höhere Kosten die Revision verursacht. Dies begünstigt das Austesten von Maßnahmen mit geringeren Revisionskosten.
  Eine zusätzliche Realoption wird nur ausgeübt, wenn dies einen positiven Erfolg im Vergleich zum Status quo erwarten lässt. Gerade bei größeren Überraschungen lassen sich vergleichsweise hohe Erfolge erzielen. Ist z. B. das Ergebnis eine lineare Funktion eines Risikofaktors, dann kann unter einfachen Bedingungen dieses Ergebnis durch Nutzung von Realoptionen zu einer konvexen Funktion verbessert werden (Franke und Hax 2009, S. 667–672).
  Die durch Periodenverkürzung entstehenden Anpassungsmöglichkeiten verbessern die Reversibilität von Maßnahmen und begünstigen damit häufig die Übernahme von mehr Risiko bereits zu Beginn des Planungszeitraumes. Dies zeigt Gollier (2001, S. 178, Prop. 42) an einem klassischen Portfolio-Problem. Unterteilt man in einem Standard-Portfolio Problem ohne Informations- und Transaktionskosten bei bekannten Wahrscheinlichkeiten einen vorgegeben Zeitraum in Perioden, an deren Beginn die Anlagepolitik jeweils angepasst werden kann, (ein statisches Modell wird durch ein flexibles ersetzt), dann wächst das optimale Portfoliorisiko, das zu Anfang des Planungszeitraums genommen wird. Denn der Entscheidungsträger kann später zwischendurch das Portfoliorisiko gemäß seinem inzwischen erreichten Vermögen anpassen und damit unerwünschten Entwicklungen seines Vermögens besser vorbeugen. Flexible Planung erlaubt daher, höhere Risiken zu nehmen.
- Die optimale Periodenlänge hängt auch von der Ausgestaltung der Anpassungsstrategie ab. Um die Verwaltungskosten von Anpassungen zu vermindern, kann der Entscheidungsträger seine Strategie standardisieren. Besonders einfach sind im Portfolio-Beispiel sog. Constant Proportion Portfolio-Strategien^23^: Hierbei ist der Anteil des Vermögens, den der Entscheidungsträger in Aktien (also riskant) anlegt (seine Aktienquote), im Zeitablauf konstant. Wenn er also Geld verloren hat, wird er seine absolute Position in Aktien vermindern, im umgekehrten Fall erhöhen. Der Vorteil dieser einfachen Strategie besteht darin, dass sich die Informationsbeschaffung jeweils auf das vorhandene Portfolio-Vermögen beschränkt und lediglich der in Aktien investierte Betrag angepasst wird.
- Wird die Zahl der Perioden durch Verkürzung erhöht, dann muss der Organisationsprozess zur Auswertung von Informationen, Entscheidungsfindung und Implementation häufiger abgewickelt werden. Dies bindet zusätzliche Personalressourcen. Auf der anderen Seite kann infolge der häufigeren Revision mit der Möglichkeit der Fehlerkorrektur der Informations- und Entscheidungsprozess vereinfacht werden. Insoweit können weniger qualifizierte Personen eingesetzt werden. Dies gilt auch, wenn eine einfachere Anpassungsstrategie gewählt wird.
  Aufgrund der diversen Einflussfaktoren und der diversen Möglichkeiten, die Corporate Governance bei einer Periodenverkürzung umzugestalten, bleibt zu klären, wie sich Kosten und Nutzen verschiedener Varianten verändern und welche Periodenlänge sich am besten eignet.

Mit der Wahl der Periodenlänge verbunden ist die schwierigere Frage, wie lang der Planungshorizont des flexiblen Modells und gleichzeitig der Rhythmus für eine revolvierende Planung gewählt werden sollen. Bei revolvierender Planung wird die gesamte Planung in vorgegebenen Zeitabständen wiederholt und dem jeweils neuesten Informationsstand angepasst. Wird häufiger revolvierend geplant und gleichzeitig der Planungszeitraum des Modells verkürzt, dann werden allerdings langfristige Konsequenzen einer Entscheidung im Modell partiell ausgeblendet, soweit sie nicht pauschaliert abgebildet werden. Je häufiger revolvierend bei kürzerem Planungshorizont geplant wird, desto mehr nähert sich die Unternehmensstrategie einem Trial and Error-Prozess. Einen vermuteten Zusammenhang zwischen Informationsqualität und Planungssystem beschreibt

#### These 8:

*Bei Verschlechterung der Informationsqualität wächst die optimale Zahl der Perioden. Gleichzeitig wird es günstiger, den Planungszeitraum des Modells abzukürzen und häufiger revolvierend zu planen*.

Der erste Teil der These beruht darauf, dass bei schlechterer Informationsqualität mehr Information pro Zeiteinheit zufließt, die erfolgreicher genutzt werden kann, weil über mehr Perioden eine raschere Reaktion ermöglicht wird. Zum zweiten Teil: Bei schlechterer Qualität wird es schwieriger, fundiert längerfristig zu planen, so dass solche Planungen öfter umgestoßen werden. Daher lohnt sich eine längerfristige Planung wegen ihrer zusätzlichen Kosten weniger, eine häufigere Neuauflage der Planung erscheint vorteilhaft.

Vor etwa 30 Jahren sagte Ulrich Weiß, Mitglied des Vorstands der Deutschen Bank, in einem Vortrag zur Strategie der Bank im entstehenden europäischen Binnenmarkt: Über eine Strategie grundsätzlich zu entscheiden beanspruche etwa 5 % Arbeitszeit, 95 % würden danach darauf verwendet, die Ergebnisse einer zunächst gewählten Vorgehensweise laufend zu ermitteln und dann die Vorgehensweise entsprechend anzupassen.

Der Hinweis, dass die grundsätzliche Entscheidung über eine Strategie lediglich 5 % Arbeitszeit in Anspruch nimmt, deutet darauf hin, dass das zu Beginn verwendete Entscheidungsmodell recht einfach ist. Dafür wird viel Arbeitszeit in die Gestaltung und Abwicklung eines Trial and Error-Prozesses investiert. Dies legt die Vermutung nahe, dass es bei schlechter Informationslage wenig Sinn macht, in die anfängliche Entscheidung viel Zeit zu investieren, sondern dass es besser ist, den Informationsprozess und den Prozess anschließender Adaptierung operativer Entscheidungen bei Sicherung der Anpassungsfähigkeit sorgfältig zu gestalten.

Diese Überlegungen erscheinen plausibel, wenn es um den Aufbau neuer Geschäftsfelder, ausgehend von wenig Information, geht. Sind ähnliche Konzepte auch geeignet, um nicht-finanzielle Risiken zu managen? Die vorangehenden Überlegungen sollen an Beispielen aus dem Bereich nicht-finanzieller Risiken eines Kreditinstituts veranschaulicht werden, dem Compliance Risiko, dem Cyber-Risiko, dem Geldwäscherisiko und dem Nachhaltigkeitsrisiko.

## Beispiele nicht-finanzieller Risiken

### Zum Management von Compliance-Risiken

Compliance-Risiken im engeren Sinn sind Risiken aus dem Verhalten von Mitarbeitern, das gegen rechtliche Regeln verstößt. Hierbei wird der Maßstab für Compliance-Risiken dem Unternehmen von außen vorgegeben. Im weiteren Sinn umfassen Compliance-Risiken auch Risiken aus Verhalten von Mitarbeitern, das gegen unternehmensinterne Verhaltensvorschriften und -kodizes verstößt. Dazu gehören z. B. Verstöße gegen intern vorgegebene Risikolimite^24^ wie auch Verstöße gegen intern vorgegebene Grundsätze ethischen Verhaltens. Dabei gibt es eine Grauzone, die aus dem Konflikt zwischen dem Ziel des Unternehmens, Überschüsse zu erwirtschaften, und ethischen Anforderungen herrührt. Dieser Konflikt wird verschärft, wenn ein Mitarbeiter an den Überschüssen des Unternehmens, die er erwirtschaftet, finanziell partizipiert. So wirft ein vorläufiger Bericht des Verkehrsausschusses des US-Repräsentantenhauses dem Flugzeughersteller Boeing vor, dass die tradierte strenge Sicherheitskultur in jüngerer Zeit durch finanzielle Anreize für die Mitarbeiter und das Streben nach hohen Aktienrenditen verwässert wurde (US-Kongress 2020). Das Management von Compliance-Risiken beinhaltet nicht nur ein Management von Risiken, sondern auch von Ertrag. Zahlreiche Bankskandale verdeutlichen diesen Konflikt. Hunt (2020) weist darauf hin, dass Verhaltensrisiken nicht nur aus Verstößen gegen Vorschriften resultieren, sondern ebenso aus dem Unterlassen erwünschten Verhaltens, z. B. einer unterlassenen Untersuchung überraschend hoher Erträge.

Fehlverhalten von Mitarbeitern kann diverse Kosten des Unternehmens verursachen, aber auch persönliche Kosten des Mitarbeiters. Wenn er gegen rechtliche Vorschriften verstößt, kann er selbst dafür mit finanziellen Bußen, Arbeitsplatzverlust und sogar mit Gefängnis bestraft werden, wie es in jüngerer Zeit häufiger geschieht.

Für das Management von Compliance-Risiken stellen sich zahlreiche Fragen, u. a.:

1. Inwieweit sollte ein Unternehmen ethische Verhaltensvorschriften erlassen, die den Unternehmenserfolg beeinträchtigen können?
2. Reicht für die Compliance-Sicherung das klassische Modell der Verteidigungslinien, bei dem die erste Verteidigungslinie direkt dem Mitarbeiter und seinen unmittelbaren Kollegen übertragen wird, die zweite Verteidigungslinie einem höher angesiedelten Risk Office, das auch für eine Gesamtschau verschiedener nicht-finanzieller Risiken verantwortlich ist, und drittens einer unabhängigen Revisionsabteilung? Oder sollten Mitarbeiter zusätzlich (z. B. anhand von elektronischen Systemen) detailliert überwacht werden? Solche Überwachungssysteme lassen sich dezentral am Arbeitsplatz installieren. Sie sind vermutlich effektiver als zentrale Überwachungsmechanismen, bedürfen allerdings der Zustimmung des Betriebsrats, da die Privatsphäre des Arbeitnehmers tangiert wird. Genügt eine unabhängig agierende Revisionsabteilung, um Führungskräfte (Beispiel Dieselskandal) zu überwachen?
3. Wie lässt sich eine nachhaltige Risikokultur im Unternehmen verankern? Da bisher Definition und Messung von Risikokultur auf erhebliche Schwierigkeiten stoßen, ist eine Nachprüfbarkeit der Effekte bisher nur eingeschränkt möglich^25^. Damit zusammen hängt auch die Steuerung des Risikoappetits der Mitarbeiter über vorzugebende Entscheidungsregeln und Anreizsysteme. Entscheidungsregeln sind bei geringer Information kaum quantitativ formulierbar, sondern eher qualitativ. Dies eröffnet erhebliche diskretionäre Spielräume. Um deren Nutzung im Interesse des Unternehmens zu sichern, gilt es, einerseits das Verhalten des Mitarbeiters durch Restriktionen einzugrenzen, um Fehlverhalten auszuschließen, und andererseits, ihm die Zielvorstellungen des Unternehmens möglichst klar zu machen, damit er demgemäß entscheiden und handeln kann. Gerade bei dürftiger Information verfügt er häufig über die beste Information „vor Ort“, so dass er gezielter entscheiden kann, gleichzeitig legen die durch Informationsmangel verschärften Agency-Probleme eine intensivere Überwachung nahe. Je weniger verlässliche Information vorhanden ist, umso mehr organisatorische Vorkehrungen sind gemäß These 1 angeraten, um Verhaltensproblemen vorzubeugen. Dazu können auch Einschränkungen der finanziellen Anreize gehören.
4. Soll das aus Fehlverhalten resultierende Reputationsrisiko zentral oder dezentral gemanagt werden? Soweit es um die Reputation des Unternehmens in der Öffentlichkeit geht, liegt ein zentrales Reputationsmanagement nahe, um die Corporate Identity in der Öffentlichkeit nachhaltig zu verankern^26^. Das Reputationsmanagement wird erschwert, wenn das Unternehmen einen Stakeholder Approach verfolgt. Jede Stakeholder-Gruppe nimmt die Reputation gemäß ihren eigenen Interessen war. Z. B. messen die Kunden die Reputation auch an der Qualität des Kundenservice, während die Gesellschafter das finanzielle Ergebnis im Auge haben. Reputationsmanagement ist daher auch Konfliktmanagement. Zwar spricht dies für ein zentrales Reputationsmanagement, aber Serviceschwächen sollten dezentral behoben werden.

Inwieweit das Unternehmen in Reputationsmanagement investieren soll, ist nur schwer zu beantworten. Zwar mögen die Kosten zusätzlichen Reputationsmanagements abschätzbar sein, aber die erzielbaren Reputationserträge sind kaum messbar; das gilt auch für vermiedene Reputationskosten, mit Ausnahme von messbaren Marktwertverlusten. Denn die Höhe der Reputationskosten kann durch das Management des Unternehmens nach Eintritt des Schadensfalls beeinflusst werden, z. B. indem es mit geschädigten Kunden einen Ausgleich sucht. Die Schätzung von Reputationskosten in Form eines Barwerts zukünftiger Ergebnisminderungen ist schwierig, weil die Vergessensrate der Stakeholder schwer vorherzusagen ist. Sie hängt nicht nur vom Management des Unternehmens ab, sondern auch davon, inwieweit Konkurrenzunternehmen von ähnlichen Schadensereignissen betroffen sind und ob andere Ereignisse, z. B. in der Politik, die Aufmerksamkeit der Stakeholder absorbieren. Es ist daher auch problematisch, Reputationskosten an unmittelbar eintretenden Marktwertverlusten des Unternehmens, wie sie im Aktienkurs zum Ausdruck kommen, zu messen. Diese überschätzen vermutlich die mittel- und langfristigen Effekte. Dies mag auch die hohen Reputationskosten erklären, die Kamiya et al. (2020) finden. Macey (2013) verdeutlicht an zahlreichen Beispielen The Death of Corporate Reputation, also das Schwinden von Reputationseffekten. Gleichzeitig weist er auf die zunehmende Regulierungsdichte hin. Es scheint, als ob die Zunahme der Regulierung mit einer Abnahme von Reputationskosten einhergeht.

Diese Überlegungen verdeutlichen, dass das Compliance Management zahlreiche offene Fragen aufwirft, die weiterer Forschung bedürfen.

### Zum Management von Cyber-Risiken

Während das Compliance-Risiko vorwiegend unternehmensendogen, dezentral von Mitarbeitern verursacht wird, kann das Cyber-Risiko zwar auch durch Fehlverhalten von Mitarbeitern und technische Probleme verursacht werden, aber ebenso durch von außen getriebene Angriffe auf die IT des Unternehmens. Zweck eines Hacking kann es z. B. sein, eine Geldzahlung zu erpressen, Geld von Konten zu stehlen, Daten zu stehlen oder die Wettbewerbsposition des Unternehmens durch Rufschädigung zu schwächen.

Die Vielfalt von IT-Problemen lässt unterschiedliche Wirkungen auf Unternehmen erwarten, dementsprechend variieren auch intern anfallende direkte Kosten, z. B. aus Wiederinstandsetzung des IT-Systems, Raub finanzieller Mittel oder Lösegeldzahlungen, und Reputationskosten. In einer Stichprobe von Cyber-Attacken auf Industrieunternehmen finden Kamiya et al. (2020), dass die direkten Kosten (out of pocket cost) im Durchschnitt lediglich etwas mehr als 1 % des gesamten Marktwertverlustes ausmachen.

Das Management von IT-Risiken wird durch die geringe verfügbare Information erschwert. Zwar werden Daten über Ausfälle von IT Systemen und daraus resultierende Schäden weltweit gesammelt, dennoch dürfte es schwierig sein, auf dieser Basis das Cyber-Risiko eines Unternehmens verlässlich abzuschätzen. Die Dunkelziffer ist vermutlich hoch, die berichteten Fälle und Schäden können einen Reporting-Bias aufweisen. Außerdem beeinflussen unternehmensspezifische Faktoren das Cyber-Risiko^27^. Kommt es nicht zu einem Ausfall des IT-Systems, dann wird das Hacking eventuell im Unternehmen gar nicht und damit auch von der Öffentlichkeit nicht bemerkt. Dann entfallen messbare Kosten.

Der Informationsmangel zu IT-Risiken legt es nahe, externe Expertise zu nutzen. Dazu bietet sich ein Outsourcing von IT-Leistungen an. Es erlaubt, Informations- und andere Spezialisierungsvorteile und economies of scale-Effekte von IT-Firmen zu nutzen und dadurch Leistungs- und Kostenvorteile zu erzielen. Es entbindet das Unternehmen allerdings nicht davon, die Qualität der outgesourcten IT-Leistungen ständig zu überprüfen.

Das betriebsinterne Management von IT-Risiken wird dadurch erschwert, dass Mitarbeiter diese anscheinend oft unterschätzen (Deloitte Deutschland 2019). Dies mag durch eine hohe Dunkelziffer von IT-Pannen getrieben sein. Der Bewusstseinsbias von Entscheidungsträgern kann dadurch verstärkt werden, dass zwar die Kosten von betrieblichen Maßnahmen zur Abwehr von IT-Risiken in der Rechnungslegung sichtbar werden, aber nicht deren unsichtbare Erträge aus verhinderten IT-Pannen. Informationsmangel und Bewusstseinsbias können dazu führen, dass IT-Vorsichtsmaßnahmen nur in geringem Umfang durchgeführt werden, so insbes. bei mittelständischen Unternehmen (Deloitte Deutschland 2019).

Zur Milderung des Informationsdefizits bietet es sich an, die Expertise der Lieferanten von Hard- und Software zu nutzen, aber auch bei Abschluss einer Cyber-Versicherung die Expertise des Versicherers, der im Allgemeinen vor Abschluss des Vertrags auf ex ante- und ex post- Maßnahmen bestehen wird, um potentielle Schäden zu reduzieren (These 6).

Die Herausforderung für das Management besteht darin, in Anbetracht von wenig verfügbarer Information und Bewusstseinsbias ein Maßnahmenpaket zu planen, das Risiko und Ertrag optimiert. Kann ein „optimales“ Investitionsvolumen in Hard- und Software nach Bauchgefühl des Vorstands bestimmt werden? Kann das IT-Management sequentiell in verschiedenen Schritten aufgebaut werden, um gezielt die betriebsinterne Information zu verbessern und gleichzeitig ein verlässliches Funktionieren der IT zu sichern? Unter welchen Bedingungen soll ein Unternehmen IT-Pannen verheimlichen, um Reputationskosten zu vermeiden?

Die mangelnde Messbarkeit von Erträgen des IT-Managements wirft auch die Frage nach geeigneten Anreizsystemen auf. Wenn ein Unternehmen mehr Geld für IT-Sicherheit aufwendet, senkt dies den Überschuss und schmälert damit sichtbar die Basis für finanzielle Anreize, während die Erträge kaum sichtbar sind. Dies begünstigt Unterinvestitionen in die IT-Sicherheit.

In Anbetracht der verschiedenen Ursachen von IT-Pannen und verschiedenen Informationsständen von Mitarbeitern stellt sich zudem die Frage, inwieweit die Unternehmensorganisation zum Management von IT-Risiken differenziert werden soll. Es liegt nahe, das von außen getriebene Cyber-Risiko zentral zu managen, IT-Verhaltensrisiken von Mitarbeitern indessen über das Compliance Management. Sind damit unterschiedliche Konzepte von Verteidigungslinien vorgezeichnet?

### Zum Management von Geldwäsche-Risiken

In den letzten Jahren ist Geldwäsche immer stärker in den Fokus von Politikern und Regulatoren geraten. Geldwäsche kann dazu dienen, aus kriminellen Aktivitäten wie z. B. Steuerhinterziehung, Drogenhandel, Erpressung, Korruption, Prostitution und Menschenhandel erworbenes Geld reinzuwaschen. Nicht selten wird versucht, schmutziges Geld bei einem Kreditinstitut einzuzahlen und dieses zu beauftragen, das Geld dann über eine Kette von Zahlungstransaktionen an einen endgültigen Zahlungsempfänger zu transferieren. Jede Bank ist daher bei einer größeren Einzahlung auf ein bei ihr geführtes Konto verpflichtet, die Quelle des Geldes und den Leumund des Einzahlers, der nicht selten ein Strohmann ist, nachzuprüfen. Nur nach gründlicher Prüfung darf die Bank die Einzahlung annehmen. Ebenso sind die Banken gehalten, die Empfänger von Zahlungen zu prüfen und ob das Geld für kriminelle Aktivitäten genutzt werden soll. Kompliziert wird die Prüfung dadurch, dass die Banken nicht nur den direkten Empfänger einer Zahlung überprüfen sollen, sondern auch den endgültigen Empfänger, der evt. über eine Kette von Mittelsmännern und zwischengeschalteten Banken das Geld empfängt. Für die Unbedenklichkeit genügt nicht, dass die Zahlung an eine bekannte Korrespondenzbank weitergeleitet wird.

Auch dürfen die Banken nicht mit Unternehmen und Personen Geschäfte machen, die von der Europäischen Union oder den USA mit entsprechenden Sanktionen belegt sind. Noch komplexer wird das Problem durch den engen Zusammenhang zwischen Korruption und Geldwäsche, den das US-Justizministerium postuliert (Hardy 2020). Selbst wenn ein Unternehmen nicht wegen Korruption bestraft werden kann, so kann es ggf. trotzdem für geleistete Zahlungen wegen Geldwäsche verurteilt werden.

Für eine Bank ist es aufwändig, die Unbedenklichkeit ihrer Geschäftspartner zu prüfen (know your customer). Eine Erleichterung schaffen gesetzlich vorgeschriebene Transparenzregister, in denen zahlreiche Angaben über Unternehmen und ihre wirtschaftlich Berechtigten zu veröffentlichen sind^28^. Stimmen die Angaben eines Kunden nicht mit denen im Transparenzregister überein oder besteht ein Verdacht auf Strohmänner, dann muss die Bank dies dem Transparenzregister melden. Verdachtsmeldungen sollen jede Bank zu besonders gründlicher Prüfung veranlassen. Wird einer Bank ein Mangel an due diligence nachgewiesen, dann entstehen nicht nur direkte Kosten aus der Annullierung von Zahlungen und Strafen, sondern auch schwer abschätzbare Reputationskosten.

Erschwerend kommt hinzu: 1. Die Definition von Geldwäsche variiert von Land zu Land. 2. Trotz Transparenzregister (die zudem nicht weltweit vorgeschrieben sind) existieren für viele Einzahler und für viele Zahlungsempfänger nur unvollständige Angaben. 3. Es gibt keine Checklist, bei deren korrekter Abarbeitung die Banken vor rechtlicher Verfolgung und Kritik der Aufseher geschützt sind.

Für Banken stellt sich damit die komplexe Aufgabe, ertragreiche Zahlungstransaktionen, insbes. internationale, bei geringem Geldwäsche-Risiko abzuwickeln. Auch mögliche Cross selling-Erträge aus Zahlungstransaktionen sind zu berücksichtigen. Damit stellen sich zahlreiche Fragen: Welche Prüfungen sind bei bestimmten Arten von Transaktionen vorzunehmen? Wie intensiv sollen diese Prüfungen sein? Wie ist zu verfahren, wenn die Prüfungsergebnisse im Graubereich liegen? Welche Mitarbeiter/Gremien sollen über welche Transaktionen nach welchen Regeln entscheiden? Mit welchen anderen Instrumenten der Corporate Governance sollen Geldwäscherisiken eingeschränkt und kontrolliert werden? Wie sind diese Vorgehensweisen gemäß verfügbarer Information zu differenzieren?

Immerhin gibt es bei Geldwäscherisiken einen Vorteil im Vergleich zu Compliance- und Cyberrisiken. Zwar lässt sich eine Wahrscheinlichkeitsverteilung potentieller Schäden aus Geldwäsche kaum schätzen, wohl aber der Ertrag aus einer nicht beanstandeten Zahlungstransaktion. Eine einfache Entscheidungsregel für die Genehmigung einer Transaktion könnte prüfen, ob der Ertrag einer Transaktion größer ist als ihr Risiko, multipliziert mit einem Parameter λ, der die Risikoaversion des Managements widerspiegelt. Da es Wahrscheinlichkeitsverteilungen kaum gibt, lässt sich ein erwarteter Ertrag kaum angeben. Stattdessen könnte die Bank den Ertrag E unter der Annahme schätzen, dass die Transaktion nicht beanstandet wird (best case-Ertrag). Das Risiko aus der Transaktion könnte anhand eines Maximum Probable Loss MPL (realistic worst case) veranschlagt werden, der ggf. bei weniger verfügbarer Information höher anzusetzen wäre. Eine Transaktion wäre dann genehmigungsfähig, wenn

$$\mathrm{E}-\lambda\,\mathrm{MPL}>\mathrm{S}.$$

Rückwirkungen auf das Risiko des Bankportfolios werden beim MPL ausgeklammert. Diese könnten grob über eine Schwelle S erfasst werden, die mit dem vermuteten Zuwachs des Bankportfolio-Risikos wächst. Diese einfache Entscheidungsregel ähnelt dem Hurwicz-Kriterium. Unter welchen Voraussetzungen eine solche Regel Sinn macht, bleibt zu klären.

Eine solche Regel kann für zentrale und für dezentrale Entscheidungen in einer Bank herangezogen werden. Als Grundlage für die Planung der erforderlichen Liquiditäts- und Kapitalreserven könnte der Maximum Probable Loss aus Geldwäscherisiken dienen.

### Zum Management von Nachhaltigkeitsrisiken

Ein jüngeres nicht-finanzielles Risiko sind Nachhaltigkeitsrisiken, so z. B. Klimarisiken, aber auch ESG-Risiken und andere aus den 17 UN-Nachhaltigkeitszielen abgeleitete Risiken. Staatliche Politik und Regulatoren/Aufseher^29^ verschärfen den Druck auf Unternehmen, ihre Politik an diesen Zielen auszurichten. Da diese nahezu alle Bereiche der Unternehmenspolitik betreffen, stellt das Nachhaltigkeitsmanagement Unternehmen vor weitreichende Herausforderungen. Z. B. werden Banken angegriffen, wenn sie Kredite an Unternehmen vergeben, die fossile Brennstoffe fördern. Ratingagenturen vergeben schlechtere Ratings an solche Unternehmen. Ebenso sollen die Manager von Publikumsfonds bei ihrer Anlage auf Nachhaltigkeitsratings von Anlageinstrumenten abstellen.

Bisher besteht wenig Einigkeit über die Definition von Nachhaltigkeit. Sie ist ein mehrdimensionales Konstrukt, dessen Dimensionen und Gewichtung umstritten sind. Sind z. B. Unternehmen nachhaltig, die Waffen produzieren? Sind startups, ein Motor für Forschung und Entwicklung, nachhaltig, obgleich ihre durchschnittliche Lebenserwartung nur wenige Jahre beträgt? Es überrascht nicht, dass ein heftiger Streit über die Definition von Nachhaltigkeit entbrannt ist. Denn es geht auch um das allmähliche Absterben einzelner Branchen und das Aufstreben nachhaltiger Branchen mit entsprechenden Folgen für Arbeitsplätze und das wirtschaftliche Überleben von Regionen.

Zudem ist unklar, wie lange die Transformation einer Ökonomie zu mehr Nachhaltigkeit dauern wird, bevor sie in ein neues, stabileres Gleichgewicht übergeht. In beiden Phasen sind die Anforderungen an Nachhaltigkeitsmanagement unterschiedlich.

Bei vollständiger Information würden Banken Nachhaltigkeitsrisiken in die Wahrscheinlichkeitsverteilungen von Kredit- und Marktpreisrisiken einbauen. Daran ist momentan in Anbetracht der vielen Ungereimtheiten kaum zu denken. Eine gröbere Reaktion von Banken könnte darin bestehen, Beträge und Fristigkeit der Kredite an nachhaltigkeitsgefährdete Unternehmen zu kürzen. Damit trägt die Bank eventuell zur Verschlechterung der Qualität bereits von ihr vergebener Kredite bei. Bei der Wertpapieranlage steht der Manager vor der Frage, ob er die Streuung des Anlageportfolios im Interesse der Nachhaltigkeit verringern soll. Auch ist bisher unklar, ob eine nachhaltige Geldanlage höhere Erträge erwarten lässt.

Die verfügbare Information zu Nachhaltigkeitsrisiken ist noch besonders gering. Deshalb erweist sich das Nachhaltigkeitsmanagement als besonders schwierig, der Wert der Warteoption ist hoch (These 7). Soll der Vorstand eines Unternehmens daher genauere Information abwarten oder *vorläufig* Nachhaltigkeit definieren und Nachhaltigkeitsziele vorgeben? Oder soll er zunächst lediglich partielle, weniger kontroverse Elemente von Nachhaltigkeit und Teilziele vorgeben, die für alle Abteilungen des Unternehmens verbindlich sind? Sind Nachhaltigkeitskosten und -erträge genügend verlässlich abschätzbar, um sie in der längerfristigen Planung, insbes. auch neuer Geschäftsfelder, zu berücksichtigen? Oder sollte vorerst ein kürzerer Planungshorizont gewählt werden (These 8)? Wie sollte ein Nachhaltigkeits-Trial and Errror-Prozess gestaltet werden?

Infolge der ausgeprägten Unklarheiten (These 4) kommt unspezifischen Reserven in Form von Liquiditäts- und Kapitalreserven erhöhte Bedeutung zu, wenn ihre Kosten niedrig sind (These 3) und der Entscheider seine Risikoaversion erhöht (These 5). Auch Regulatoren und Aufseher stehen vor zahlreichen ungelösten Fragen. Insbes. sind externe Kosten und Erträge strengerer Nachhaltigkeitserfordernisse bisher kaum abschätzbar; daher sind auch detaillierte externe Reservevorgaben problematisch.

## Herausforderungen für Praxis und Hochschule

### Praxis

Die vier Beispiele nicht-finanzieller Risiken verdeutlichen die Probleme, die geringe verfügbare Information für die Corporate Governance aufwirft. Vor ähnlichen Problemen standen Wirtschaft und Politik auch in der Corona-Krise: Ihr Höhepunkt und weiterer Verlauf waren anfangs kaum prognostizierbar. In zahlreichen Staaten reagierte die Politik mit einer Art von max-min Strategie: Das öffentliche Leben, die private Freiheit und die Wirtschaftstätigkeit wurden bis an die Grenze des Erträglichen eingeschränkt in der Hoffnung, damit das langfristige Wohlergehen zu maximieren.

Nicht-finanzielle Risiken unterscheiden sich hinsichtlich diverser Merkmale: Inwieweit sind sie exogen/endogen, zentral/dezentral verursacht, inwieweit gibt es Informationen zu den kurz-/mittel- und langfristigen Wirkungen einzelner Risiken? Welche anderen Merkmale sind für das Management dieser Risiken relevant? Dazu tritt die noch schwierigere Aufgabe, Ertrag und Risiko von Maßnahmen zum Management einzelner Risiken zu prognostizieren.

Die Unterschiede zwischen den nicht-finanziellen Risiken werfen die Frage auf, inwieweit a) Planung und Entscheidung sowie b) Steuerung und Kontrolle dieser Risiken einheitlich oder differenziert gestaltet werden sollen. Beides greift eng ineinander und bedarf daher gemeinsamer Gestaltung. Die übliche Trennung von Entscheidungs- und Organisationsforschung wird den Herausforderungen nicht-finanzieller Risiken nicht gerecht.

Damit zusammen hängt die Frage, ob die Verantwortung für finanzielle und nicht-finanzielle Risiken bei einer Person im Vorstand gebündelt werden soll. Einerseits liegt dies nahe, weil das Wohlergehen eines Unternehmens auch von den Portfolioeffekten dieser Risiken abhängt, andererseits sind diese Risiken so unterschiedlich, dass eine Person mit ihrem Verständnis überfordert ist. Wird die Verantwortung für einzelne Risiken verschiedenen Personen zugeordnet, dann muss die Corporate Governance anderweitig die Kontrolle der Portfolioeffekte sicherstellen.

Mit den Kontrollfragen eng zusammen hängt die Gestaltung von Risikoberichten. In Banken soll sich der Vorstand täglich ein Bild über das Risiko der Bank machen. Vielfach wünscht der Vorstand, das Risiko einer Bank in wenigen Zahlen zusammenzufassen, um rasch erkennen zu können, ob vorgegebene Risikolimite eingehalten werden. Diese Vorgehensweise mag bei finanziellen Risiken gerechtfertigt sein, jedoch kaum bei nicht-finanziellen Risiken. Hunt (2020), der selbst Regulator, Aufseher und Risikomanager war, spricht von der dashboard illusion. Für einzelne nicht-finanzielle Risiken wird eine Messbarkeit in Zahlen unterstellt, die es so nicht gibt. Selbst wenn Messbarkeit gegeben wäre, bräuchte es Leser, die die Zahlen verstehen. Wie sollte ein Reporting aussehen? Diese Problematik stellt sich auch für nicht-finanzielle Unternehmen.

In Anbetracht der geringen Informationsqualität liegt es nahe, die Informationsbeschaffung erheblich zu intensivieren. Die zunehmende Fülle an elektronisch verfügbarer Information suggeriert, mit Big Data-Methoden all diese Informationskanäle zu nutzen. Hierbei sind indessen zwei Trugschlüsse zu vermeiden. Erstens schleichen sich in die Big Data zunehmend Fake News ein, die immer besser getarnt werden. Dies wirft die schwierige Aufgabe auf, verlässliche von nicht verlässlichen Informationen zu trennen. Zweitens zeigt die statistische Analyse von Big Data oft signifikante Zusammenhänge, deren Prognosegehalt gering ist. Für Out of Sample-Tests fehlen häufig die erforderlichen längeren Beobachtungszeiträume. Inwieweit künstliche Intelligenz bei beiden Probleme Abhilfe schaffen kann, bleibt weiterer Forschung vorbehalten.

### Hochschule

Die Vielfalt der Fragen, die das Management von nicht-finanziellen Risiken aufwirft, stellt die Unternehmen vor große Herausforderungen. Daher erwarten sie von den Hochschulen Unterstützung. Forschern bieten sich damit neue Forschungsfelder, deren Ergebnisse entgegen manchen Einschätzungen auch Eingang in erstklassige Zeitschriften finden.

Wie könnte ein Forschungsansatz aussehen? In Anbetracht der geringen Information zu nicht-finanziellen Unternehmensrisiken erzeugt diesbezügliche Forschung ein nicht-finanzielles Risiko der Hochschule. Dies legt auch ein anderes als das übliche Forschungsdesign nahe. Da es um die Verknüpfung von finanzwirtschaftlicher Planung und Organisationskonzepten geht, bietet sich eine Kooperation zwischen Forschern beider Bereiche an, um Synergie-Effekte zu erzielen. In einem ersten Schritt wird dieses Forschungsteam sein Informationsdefizit zu nicht-finanziellen Risiken durch intensive Gespräche mit einschlägigen Praxisvertretern abbauen. Diese können den Hochschulvertretern am besten erklären, welcher Art die relevanten nicht-finanziellen Risiken sind und welche Fragen ihr Management aufwirft. Diese Information ist für eine Forschung, die sich nicht im Elfenbeinturm bewegt, unerlässlich.

Sodann beginnt die eigentliche Forschungsarbeit. Da es „Forschungsirrläufer“ geben kann, ist die Einrichtung von Verteidigungslinien erforderlich. Eine erste Verteidigungslinie könnte im Forschungsteam selbst geschaffen werden, indem regelmäßig und systematisch Stärken und Schwächen des eigenen Ansatzes auf den Prüfstand kommen. Eine zweite Verteidigungslinie könnte die Präsentation der Ergebnisse vor Forschern sein, die Planungs‑/Entscheidungsforschung bzw. Organisationsforschung „klassisch“ betreiben. Eine dritte Verteidigungslinie wäre das kritische Feedback von Praxisvertretern. Eine vierte Verteidigungslinie bieten schließlich Begutachtungsprozesse, bereitgestellt von einschlägigen Konferenzen und Zeitschriften. Wichtig erscheint es, die Flexibilität der Forschungsstrategie zu sichern, damit „Forschungsirrläufer“ frühzeitig zu erträglichen „Kosten“ korrigiert werden können. Es ist zu hoffen, dass sich genügend Forscher an Hochschulen dieser Herausforderung stellen.

## Anhang A

### Lösungsansatz bei Absatzrisiko

Da die Absatzmenge stochastisch ist, wird vereinfachend ein konstanter Absatzpreis *p* unterstellt. Existieren Terminkontrakte auf den Wechselkurs, so kann der Exporteur z. B. den erwarteten Exporterlös in Fremdwährung durch ein Termingeschäft absichern. Dann bleibt allerdings ein Wechselkursrisiko aus der Differenz von tatsächlichem und abgesichertem Fremdwährungserlös. Was bedeutet dies für die optimale Produktionsmenge des Exportgutes?

Der Cashflow im Zeitpunkt 1 ist bei kostenloser Entsorgung nicht verkaufter Einheiten gleich

3 $$\begin{aligned} \mathrm{C}\left(\mathrm{x},\mathrm{y}\right) & =-K_{f}+\mathrm{p}\mathrm{w}\mathrm{X}-\mathrm{k}(\mathrm{x})\mathrm{x}-(\mathrm{w}-\mathrm{f})\mathrm{y}\\ & =-K_{f}+(\mathrm{p}\mathrm{f}-\mathrm{k}(\mathrm{x}))\mathrm{x}+(\mathrm{p}\mathrm{x}-\mathrm{y})(\mathrm{w}-\mathrm{f})-\mathrm{p}\mathrm{w} \left(\mathrm{x}-\mathrm{X}\right)^{+}, \end{aligned}$$

$$\text{wobei}\,\left(\mathrm{x}-\mathrm{X}\right)^{+}=0,\text{falls}\,\mathrm{x}<\mathrm{X},\mathrm{und}\, \left(\mathrm{x}-\mathrm{X}\right)^{+}=\mathrm{x}-\mathrm{X},\text{ falls}\,\mathrm{x}\geq \mathrm{X}.$$

Der Cashflow C(x,y) ist gleich dem Cashflow (1) bei bekannter Absatzmenge minus dem aus Nichtverkauf produzierter Einheiten entgangenen Erlös *p* w (x −X)^+^. Dieser ist ein Produkt von zwei stochastischen Variablen.

Bei umfassender Information ist die gemeinsame Wahrscheinlichkeitsverteilung von X und w bekannt. Vereinfachend seien Wechselkurs und Absatzmenge unabhängig voneinander verteilt. Dann bietet es sich an, das Absatzrisiko als multiplikatives Hintergrundrisiko mit negativem Erwartungswert zu modellieren (siehe auch Adam-Müller 1995, Kap. 3.1). Jede Kombination aus Absatzmenge und Wechselkurs definiert einen Zustand der Natur z(X,w) mit dem zugehörigen bedingten Cashflow C(x,y|X,w) und dem Nutzen U[C(x,y|X,w)]. Bildet man bei gegebenem Wechselkurs den Erwartungswert über die Absatzmenge X, so definiert dieser die indirekte Nutzenfunktion U*[C(x,y|w)]. Die Produktionsmenge x und das Hedgevolumen y werden sodann optimiert, indem der über den Wechselkurs gebildete Erwartungswert der indirekten Nutzenfunktion maximiert wird.

Wie beeinflusst das Hintergrundrisiko die Entscheidungen? Im Vergleich zur Situation ohne Absatzrisiko senkt die „Strafe“ – *p* w (x − X)^+^ bei gegebenem Hedgevolumen die optimale Produktionsmenge, weil sich so die Strafe senken lässt. Unterstellt man wie üblich eine Nutzenfunktion mit abnehmender absoluter Risikoaversion, dann erhöht die Strafe zudem die Risikoaversion in den Strafzuständen. Auch dies vermindert die Produktionsmenge. Je nach indirekter Nutzenfunktion kann das Absatzrisiko diverse Effekte auf das optimale Hedgevolumen erzeugen. Hier kann es zudem zu cross hedging-Effekten aus dem Hedgeergebnis (*p* x − y) (w − f) und der Strafe – *p* w (x − X)^+^ mit Rückwirkungen auf die optimale Produktionsmenge kommen. Bereits im Newsboy Problem, in dem es kein Wechselkursrisiko gibt, hängen Antworten der komparativen Statik von der Nutzenfunktion ab (Eeckhoudt et al. 1995). Das gilt erst recht bei multiplikativem Hintergrundrisiko.

## Anhang B

### Intervalle bei qualitativen Wahrscheinlichkeiten

Gegeben seien die Ereignisse i = 1,..,*n*, wobei Ereignis i mindestens ebenso wahrscheinlich ist wie Ereignis i‑1. Bezeichnet Φ_i_ die qualitative Wahrscheinlichkeit von Ereignis i, dann gilt Φ_i_ ≥ Φ_i−1_, i > 1. Konsistent mit diesen Wahrscheinlichkeiten existieren quantitative Wahrscheinlichkeiten π_i_ mit π_i_ ≥ π_i−1_, i > 1. Die minimale quantitative Wahrscheinlichkeit eines Ereignisses ist gleich 0 für i = 1,..,*n* − 1, so dass die maximale Wahrscheinlichkeit des Ereignisses *n* gleich 1 ist. Die maximale quantitative Wahrscheinlichkeit eines Ereignisses i ist gleich 1/(*n* − i + 1) für i = 1,..,*n* − 1, nämlich wenn alle Ereignisse j ≥ i gleich wahrscheinlich sind und alle Ereignisse j < i eine Wahrscheinlichkeit von 0 haben. Daraus folgt für das Intervall ∆_i_, in dem die quantitative Wahrscheinlichkeit des Ereignisses i liegt,

$$\Updelta _{i}=1/(\mathrm{n}-\mathrm{i}+1)\text{ f\"{u}r }\mathrm{i}=1,\ldots,\mathrm{n}-1 \mathrm{und} \Updelta _{n}=1-1/\mathrm{n}.$$

Die Wahrscheinlichkeit des Ereignisses *n* ist minimal bei Gleichverteilung aller Ereignisse.

Das Wahrscheinlichkeitsintervall wächst mit i, also mit höherer qualitativer Wahrscheinlichkeit, ausgehend von einem sehr niedrigen Wert bei hohem n; es erreicht die Größe 1/2 für das zweitwahrscheinlichste Ereignis und fast die Größe 1 für das wahrscheinlichste Ereignis. Das Intervall eines Ereignisses, ausgenommen das wahrscheinlichste, wächst, wenn die Zahl der Ereignisse vermindert wird. Sofern nur wenig wahrscheinliche Ereignisse den Value at Risk oder den Expected Shortfall und damit die Reservebildung bestimmen, spielen die Intervalle hierfür nur eine geringe Rolle. Ertrag und Risiko operativer Handlungsalternativen hängen allerdings von allen Ereignissen ab, so dass die Intervalle eine große Rolle spielen können.

## Footnotes

1 So wurden zahlreichen Banken von Gerichten, Aufsichtsbehörden und Justizbehörden hohe Strafen wegen Fehlverhaltens auferlegt (Golin und Moses 2019), so bei Verbriefung von Kreditrisiken (US Department of Justice 2014, fondsprofessionell 2018), ebenso wegen Steuerhinterziehung oder Hilfe dabei (Iseli 2018; swissinfo 2019). Ähnliches gilt für unzulässige Absprachen von Bankmitarbeitern zur Manipulation von Wechselkursen (Handelszeitung 2015) und Zinssätzen (Handelszeitung 2019).

2 Zu erheblichen Strafen wegen Geldwäsche siehe Osman (2019).

3 So stufen Ratingagenturen inzwischen Unternehmen herab, die den Klimaschutz gefährden (Morningstar 2019), z. B. Unternehmen, die fossile Energie fördern oder nutzen. Auch werden Unternehmen herabgeratet, deren Corporate Governance als schwach und daher nicht nachhaltig eingestuft wird. Für die Banken bedeutet dies, dass sie bei ihrer Kreditvergabe und ihrer Geldanlage Nachhaltigkeitsrisiken in Betracht ziehen sollten.

4 Kaplan und Mikes (2012) unterscheiden Preventable Risks, verursacht durch unternehmensinterne Ereignisse und durch betriebliche Vorkehrungen verhinderbar, Strategierisiken, die durch die selbst gewählte Unternehmensstrategie erzeugt werden, und externe Risiken, z. B. aus Naturkatastrophen. Sie schlagen unterschiedliche Vorgehensweisen des Risikomanagements für diese Risikokategorien vor.

5 Zu den nicht-finanziellen Risiken zählen oft strategische Risiken, getrieben vom Verhalten der Kunden, der Wettbewerber, der Politik und des Unternehmens selbst.

6 Objektive Wahrscheinlichkeiten gibt es nur in Ausnahmefällen. Daher greifen die Entscheidungsträger mangels besserer Alternativen auf statistisch objektivierte oder subjektive Wahrscheinlichkeiten zurück.

7 Eine isolierte Bewertung einzelner geschäftlichen Aktivitäten wäre nur bei Wertadditivität zulässig. Bei Wertadditivität ergibt sich der Wert des Unternehmens als Summe der Werte der einzelnen Aktivitäten. Dann erübrigt sich jedoch auch ein Risikomanagement.

8 Die Lagerhaltung von Rohstoffen und Fertigprodukten dient auch der unspezifischen Absicherung gegen unerwartete Lieferengpässe bei Beschaffung und Absatz.

9 Hierzu zählen neben konkreten Maßnahmen auch Ge- und Verbote. Z. B. werden bestimmte geschäftliche Aktivitäten des Unternehmens verboten, weil die davon ausgehenden Risiken als zu hoch erachtet werden. So kann eine Bank internationale Zahlungen mit Gruppen verdächtiger Zahlungsteilnehmer von vornherein verbieten. Oder es wird geboten, stets Antivirenprogramme in die IT einzubauen, um Cyberattacken zu erschweren. Oder die Höhe von Einzelkrediten wird beschränkt, um eine Mindest-Risikostreuung des Kreditportfolios zu sichern.

10 Fehler in der Hedgingpolitik können gravierende Folgen nach sich ziehen, wie z. B. 1993 die existentielle Krise der Metallgesellschaft.

11 Informations-und Transaktionskosten schlagen sich in Unvollkommenheiten des Kapitalmarkts nieder. Diese können auch zu Bewertungsverzerrungen führen. So wirft die Bewertung von Finanzoptionen nach wie vor Puzzles auf, siehe z. B. Jackwerth (2000) und Garleanu et al. (2009).

12 Bei vollkommenem Kapitalmarkt wären die Bestandshaltekosten gleich null. Allerdings sind die Kosten des Eigenkapitals und damit von Kapitalpuffern kontrovers, wie die vielfältigen Bemühungen von Banken zur Minimierung ihres Eigenkapitals verdeutlichen.

13 Hier werden nur Terminkontrakte untersucht. Können auch Optionskontrakte genutzt werden, dann kann das Portfolio von Absicherungskontrakten gemäß den Gegebenheiten des Unternehmens verbessert werden. Vgl. Brown und Toft (2002).

14 Für mehrperiodige Entscheidungsprobleme gibt es eine umfangreiche Literatur zur optimalen Produktions- und Lagerhaltung, so Sasieni et al. (1959, Ch. 4).

15 Geht man von einer HARA-Nutzenfunktion mit abnehmender absoluter Risikoaversion und relativer Risikoaversion von mehr als 1 aus, dann ist die Risikoaversion der indirekten Nutzenfunktion geringer (höher) zu als die der ursprünglichen Nutzenfunktion, wenn die relative Risikoaversion der ursprünglichen Nutzenfunktion im Vermögen steigt (sinkt). Entgegen der Intuition ist es also möglich, dass die Risikoaversion bei multiplikativem Hintergrundrisiko sinkt.

16 Ausgehend von geringer Qualität der verfügbaren Information stellt dieses Modell dennoch hohe Anforderungen an die Information. Daher dürfte nur eine mehr oder weniger pauschale praktische Umsetzung des Modellgedankens in Betracht kommen.

17 Siehe auch Izhakian und Brenner (2018).

18 Dies schließt an die bekannte Diskussion an, welche Ereignisse im Bewusstsein des Entscheiders als entscheidungsrelevant gelten und welche vernachlässigt werden.

19 Die Feinheit der Aufteilung eines Realisationsintervalls könnte sich ebenfalls an Fühlbarkeitsschwellen orientieren.

20 Siehe auch Flood und Korenko (2015), Glasserman et al. (2015). In Europa bestehen insbes. die Aufsichtsbehörden auf Stress Tests, so z. B. die European Banking Authority (2018).

21 Z. B. mag für einen Exporteur ein Kreditausfallrisiko seines Kunden in Argentinien schlecht abschätzbar und somit ein nicht-finanzielles Risiko sein. Dennoch mag es in Argentinien besser informierte Banken geben, die hierin ein finanzielles Risiko sehen und eine weitgehende Übernahme des Ausfallrisikos preiswert anbieten.

22 Dazu gehören z. B. simulierte Attacken von betriebsexternen und internen Fachleuten, um Schwachstellen des IT-Systems herauszufinden und zu beseitigen.

23 Diese Strategie ist sogar optimal, wenn es keine Informations- und Transaktionskosten gibt, die Aktienrendite einer standard-geometrischen Brownschen Bewegung gehorcht und der Entscheidungsträger konstante relative Risikoaversion hat (Merton 1971).

24 Bekannt wurden verschiedene rogue-trader, die ihrem Arbeitgeber hohe Handelsverluste bescherten, so Nick Leeson (Barings Bank) in den 90er Jahren.

25 Bannier et al. (2020) versuchen, das Risikoverhalten in Unternehmen über verschiedene Indikatoren zu messen und die Wirkung auf das Unternehmensrisiko abzuschätzen.

26 Zum Reputationsmanagement siehe auch Eccles et al. (2007) und Gaultier-Gaillard et al. (2009).

27 Vermutlich sind größere, rentable Unternehmen eher Opfer von Hacking, weil sie über mehr interessante Daten verfügen und das Erpressungspotenzial höher ist (vgl. auch Kamiya et al. 2020). Umgekehrt kann die größere Zahl von unterschiedlichen IT-Systemen in großen Häusern das Hacking erschweren. Vielleicht schützt sich ein größeres Haus aber besser.

28 In den §§ 18–26 GwG (Geldwäschegesetz) finden sich die Vorgaben für das elektronische Transparenzregister. Unternehmen müssen Angaben über ihre wirtschaftlich Berechtigten an das Transparenzregister melden. Im Transparenzregister soll im Regelfall die am Ende einer Kette stehende natürliche Person mit Kennzeichnung von Art und Umfang ihres wirtschaftlichen Interesses erfasst werden.

29 So. z. B. BaFin (2019), EBA (2019), ESMA (2020).
